# Efficiency Optimization of Magnetically Coupled Resonant WPT Systems in Seawater with Variable Conductivity

**DOI:** 10.3390/s26175323

**Published:** 2026-08-22

**Authors:** Yu Xu, Wangling Mei, Jiageng Chen, Xizheng Li, Kun Zhang, Yuyang Liu, Yue Sun, Xianjun Wu

**Affiliations:** 1 College of Physics and Electronic Engineering, Xinyang Normal University, Xinyang 464300, China; mwl24@xynu.edu.cn (W.M.); 17518681320@163.com (J.C.); lixizheng0301@163.com (X.L.); zk@xynu.edu.cn (K.Z.); yuyangliu@xynu.edu.cn (Y.L.); cathy_sy@126.com (Y.S.); 2 Xinyang Gumai Optronics Co., Ltd., Xinyang 464100, China; xygm88882023@163.com

**Keywords:** magnetically coupled resonant wireless transmission, eddy current loss, conductivity, resonance frequency, transmission efficiency

## Abstract

Magnetically coupled resonant wireless power transmission (MCR-WPT) is an ideal solution for underwater wireless power transmission (UWPT). However, due to the conductivity of seawater, eddy current loss significantly reduces system transmission efficiency. This study develops an analytical estimation method to derive explicit expressions for eddy current loss and transmission efficiency, characterize their dependence on key parameters, and analyze the resonance frequency characteristics of the MCR-WPT system under different conductivities. The optimal resonant frequency and transmission efficiency improvement under variable-conductivity underwater environments are investigated. First, the coil model is simplified to its equivalent form. Based on the Biot–Savart law, the magnetic field is calculated by integral operations, and an analytical model of the eddy current loss is formulated. Consequently, the expression for the system transmission efficiency in seawater at different depths is derived, and a specific resonance frequency is identified at which the efficiency attains its maximum value. An underwater coil model is established using Ansys Maxwell finite element analysis (FEA), and the effects of electrical conductivity and resonance frequency on eddy current loss and system efficiency are analyzed. Finally, an underwater experimental platform is constructed. A freshwater solution and seawater solutions with varying electrical conductivities are prepared using artificial sea salt and pure water; frequency-sweeping experiments are then conducted. The experimental results are in good agreement with the theoretical analysis and simulations, thereby validating the accuracy of the proposed model.

## 1. Introduction

With the growing scale of marine resource exploitation, the demand for underwater equipment has increased substantially, and the urgent challenge of power supply for such equipment remains to be addressed [1,2]. Compared with traditional wired power transmission, wireless power transfer (WPT) avoids the issues of periodic battery replacement, underwater wet-plug connections, and aging of exposed cables. It offers significant advantages in terms of safety, reliability, flexibility, and stealth [3,4,5,6], making it particularly suitable for complex seawater environments. MCR-WPT offers higher output power and transmission efficiency than microwave-based approaches, a longer transmission distance than inductive power transfer (IPT), and superior misalignment tolerance. It is therefore considered a promising solution for wireless power transfer in seawater environments [7,8,9].

MCR-WPT systems in air have been extensively studied, and mature design methods have been developed for coil structure design, theoretical modeling and analysis, compensation topologies, adaptive tuning, and frequency tracking. To address coil misalignment, Otomo Y et al. (2023) proposed a magneto-electric collaborative optimization method for the joint design of transmitting and receiving coils, enabling the optimized WPT system to maintain a transmission efficiency above 90% under misalignment conditions [10]. Rezazade S et al. (2022) analyzed the transmission efficiency characteristics of typical compensation topologies and, on this basis, designed a hybrid compensation structure to improve transmission efficiency [11]. Pahlavan S et al. (2022) proposed a two-layer star-shaped transmitter coil array with a 45° overlap, in which reverse-current excitation was employed to generate a horizontal magnetic-field component, this design enabled the receiving coil to achieve a transmission efficiency of 11.5% under a 90° rotational misalignment, whereas the conventional structure was unable to transfer power under the same condition [12]. They also compared the interconnection methods among different resonators and verified the advantages of series and parallel configurations in providing uniform power to moving targets [13].

However, the high electrical conductivity of seawater induces eddy current loss in the WPT system, which in turn significantly reduces the power transmission efficiency (PTE). The mature design methods and control strategies developed for operation in air cannot be directly applied to underwater environments. In underwater environments, previous studies have attempted to mitigate eddy current losses through the optimization of conductor geometry, spatial layout, and material properties [14,15,16,17,18,19]. However, most of these investigations have focused on a single resonance frequency and a fixed conductivity, neglecting the effects of resonance frequency and the conductivity of actual seawater on system performance. Yan Z et al. (2018) and Zhang K et al. (2016) demonstrated that the total efficiency in seawater can be decomposed into two components: the eddy current loss in seawater and the transmission efficiency in air [20,21]. Consequently, determining the transmission efficiency in seawater is essentially a matter of calculating the eddy current loss. Ref. [22] first proposed a comprehensive optimization method that integrates impedance matching with dual-ended control for underwater WPT systems, taking eddy current loss into consideration. In this method, impedance matching is indirectly achieved by regulating the primary coil current [22]. Experimental results demonstrate that the eddy current loss is reduced by 48.1%. Zhang K et al. (2018) proposed that the eddy current loss is jointly induced by the electric fields excited by the primary and secondary currents, they decomposed the total loss into Peddy1 and Peddy2, and introduced Reddy1 and Reddy2 to modify the circuit model accordingly [23]. Zhao Y et al. (2025) employed the Z-parameter analysis method to model the eddy current loss as three equivalent resistances, located at the transmitting side, the receiving side, and the mutual inductance branch, respectively [24]. Fei Y et al. (2025) also introduced the concepts of equivalent eddy current self-impedance and mutual impedance, and optimized the current distribution of the coils based on the mutual impedance to suppress the eddy current loss [25].

The above studies have made valuable contributions to reducing eddy current losses; they generally approximate the eddy current loss as an equivalent resistance for calculation purposes, which is a relatively simplified approach that requires further refinement in accuracy. To date, several studies have been dedicated to improving the accuracy of eddy current loss modeling. Qiao K et al. (2022) introduced an equivalent eddy current impedance and proposed a parameter optimization method for constant-current topologies, combined with an algorithm for system optimization to enhance transmission efficiency, experimental results show that the efficiency in seawater can reach 91.12% [26]. Sun P et al. (2022) derived the eddy current loss expression based on Maxwell’s equations and introduced an eddy current loss coefficient to calibrate the theoretical values [27]. Zhai Y et al. (2025) also derived the eddy current loss expression based on Maxwell’s equations, and introduced an attenuation factor to correct the mutual inductance [28]. Zhao Y et al. (2025) introduced an amplitude attenuation factor and phase shift, and proposed a parameter identification method based on primary-side frequency measurement [24]. This provides theoretical support for correcting the equivalent inductance and equivalent resistance of the eddy current loss. However, most of these studies approach eddy current loss from the perspective of electric field analysis, whereas research based on magnetic field modeling remains scarce. The above integral expressions are computationally complex. They do not explicitly reveal the effects of key parameters on eddy current loss. This limits, to some extent, a deeper understanding of the underlying physical relationships and rapid estimation.

Furthermore, seawater conductivity is not constant; it varies with depth and temperature [28,29,30]. Zheng Z et al. (2018) first revealed a stable three-stage vertical distribution of seawater conductivity, with temperature being a key influencing factor, they also analyzed the power-law relationship between temperature and depth, as well as the linear relationship between temperature and conductivity [31]. Xu H et al. (2025) pointed out that multi-physical field coupling (namely temperature, pressure, and salinity-collectively) influences seawater conductivity [32]. Similarly, Xiao S et al. (2023) also noted that seawater conductivity is affected by temperature [33]. Zhang K et al. (2018) concluded through theoretical analysis that higher conductivity reduces the amplitude of the electric field and significantly affects its phase, leading to higher eddy current losses [23]. Rong E et al. (2023) identified the conductivity and dielectric constant of water as key parameters affecting coupler performance, they conducted simulations and experiments in freshwater with a conductivity of 0.0002 S/m, verifying a transmission efficiency of 87.2% under this specific condition [34]. Guo Y et al. (2024) measured the salinity using a salinometer (Model 8400B), with test results showing a conductivity range of approximately 3.1 S/m to 5.2 S/m over a temperature range of 2 °C to 25 °C [35]. Most existing studies are confined to a specific conductivity or remain purely theoretical. Therefore, it is necessary to develop an eddy current loss model based on magnetic field analysis and derive an explicit expression for evaluating eddy current loss in seawater and transmission efficiency. and to investigate the influence of resonance frequency on MCR-WPT systems under variable conductivity through combined simulation and experimental studies. This will help reveal the relationship among resonance frequency, conductivity, and transmission efficiency, and is critical for further improving the performance of WPT systems in seawater environments.

Motivated by the above considerations, this study optimizes the resonance frequency of the underwater magnetically coupled MCR-WPT system to enhance transmission efficiency, providing theoretical and technical support for wireless power transfer in seawater environments. First, a general theoretical model of the MCR-WPT system is developed by integrating the magnetic field distribution. Numerical analysis is then conducted to examine the effects of resonance frequency on eddy current loss and transmission efficiency, confirming the existence of an optimal resonance frequency that maximizes the efficiency. Then, an underwater WPT coil model is established in the finite element analysis software Ansys Maxwell, and the effects of conductivity (with full immersion), transmission distance, medium type, and resonance frequency on eddy current loss and transmission efficiency are analyzed to verify the theoretical derivation. Finally, an experimental platform is constructed, and solutions with varying conductivities are prepared for validation tests. The experimental results are then compared with simulation and theoretical results to further validate the accuracy of the theoretical model.

## 2. Analysis of Wireless Power Transfer Systems

### 2.1. Analysis of WPT Systems in Air

**Figure 1:**
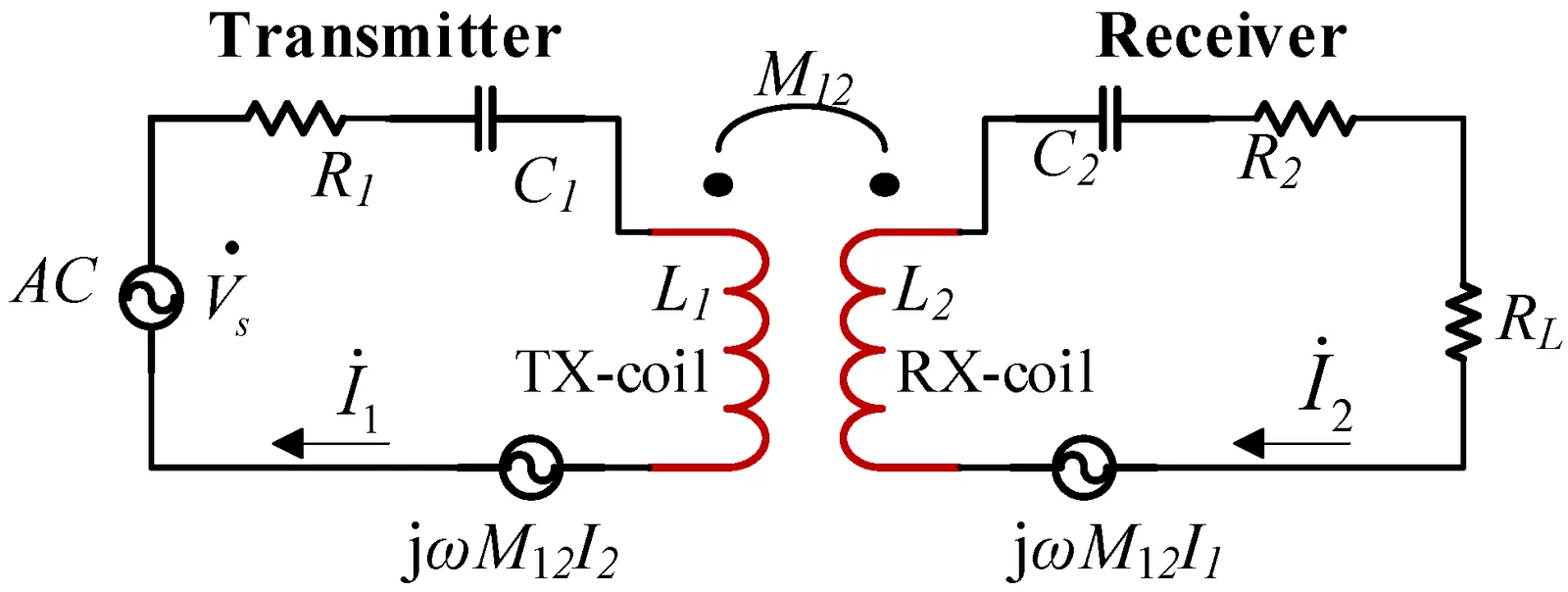
Equivalent circuit model of the MCR-WPT system in air.

Figure 1 illustrates the equivalent circuit model (ECM) of the MCR-WPT system in air. Based on Kirchhoff’s voltage law (KVL), the governing equations of the ECM can be expressed as follows:

(1) $$\left\{\begin{array}{l} {\dot{V}}_{s}=\left(R_{1}+\frac{1}{j\omega C_{1}}+j\omega L_{1}\right){\dot{I}}_{1}+j\omega M_{12}{\dot{I}}_{2}\\ 0=\left(R_{2}+R_{L}+j\omega L_{2}+\frac{1}{j\omega C_{2}}\right){\dot{I}}_{2}+j\omega M_{12}{\dot{I}}_{1} \end{array}\right.$$

For ease of expression, the equivalent impedances ${\dot{Z}}_{1}$ and ${\dot{Z}}_{2}$ of the transmitting side and the receiving side can be expressed as follows:

(2) $${\dot{Z}}_{1}=R_{1}+\frac{1}{j\omega C_{1}}+j\omega L_{1},{\dot{Z}}_{2}=R_{2}+R_{L}+j\omega L_{2}+\frac{1}{j\omega C_{2}}$$

Solving (1) yields the currents ${\dot{I}}_{1}$ and ${\dot{I}}_{2}$ of the transmitting side and the receiving side, respectively, as follows:

(3) $${\dot{I}}_{1}=\frac{{\dot{Z}}_{2}{\dot{V}}_{s}}{{\dot{Z}}_{1}{\dot{Z}}_{2}+\omega^{2}M_{12}^{2}},{\dot{I}}_{2}=-\frac{j\omega M_{12}{\dot{V}}_{s}}{{\dot{Z}}_{1}{\dot{Z}}_{2}+\omega^{2}M_{12}^{2}}$$

Power transfer efficiency (PTE) is defined as the ratio of the load power consumed by $R_{L}$ to the available power at the transmitting side. At resonance ($\omega =\omega_{0}=2\pi f_{0}=1/\sqrt{L_{1}C_{1}}=1/\sqrt{L_{2}C_{2}}$), the transmission efficiency of the MCR-WPT system in air is given as follows:

(4) ηfree=PRLPin=I˙22RLRe(V˙sI˙1)=ω02M122RLR1(R2+RL)2+ω02M122R2+RL

In this study, $P_{RL}$ denotes the load power at the receiving side, while $P_{in}$ denotes the active power at the transmitting side. The latter is measured at the transmitting-side feeding port. From (4), the partial derivative of the PTE with respect to the resonant angular frequency $\omega_{0}$ can be obtained as follows:

(5) $$\begin{array}{l} \frac{\partial \eta_{free}}{\partial \omega_{0}}=\frac{2M_{12}^{2}R_{L}\omega_{0}\left[R_{1}{\left(R_{2}+R_{L}\right)}^{2}+\omega_{0}^{2}M_{12}^{2}\left(R_{2}+R_{L}\right)\right]-\omega_{0}^{2}M_{12}^{2}R_{L}\left[2\omega_{0}M_{12}^{2}\left(R_{2}+R_{L}\right)\right]}{{\left[R_{1}{\left(R_{2}+R_{L}\right)}^{2}+\omega_{0}^{2}M_{12}^{2}\left(R_{2}+R_{L}\right)\right]}^{2}}\\ =\frac{2M_{12}^{2}R_{1}R_{L}\omega_{0}{\left(R_{2}+R_{L}\right)}^{2}}{{\left[R_{1}{\left(R_{2}+R_{L}\right)}^{2}+\omega_{0}^{2}M_{12}^{2}\left(R_{2}+R_{L}\right)\right]}^{2}}\\ =\frac{2M_{12}^{2}R_{1}R_{L}\omega_{0}}{{\left[R_{1}(R_{2}+R_{L})+\omega_{0}^{2}M_{12}^{2}\right]}^{2}}>0 \end{array}$$

**Figure 2:**
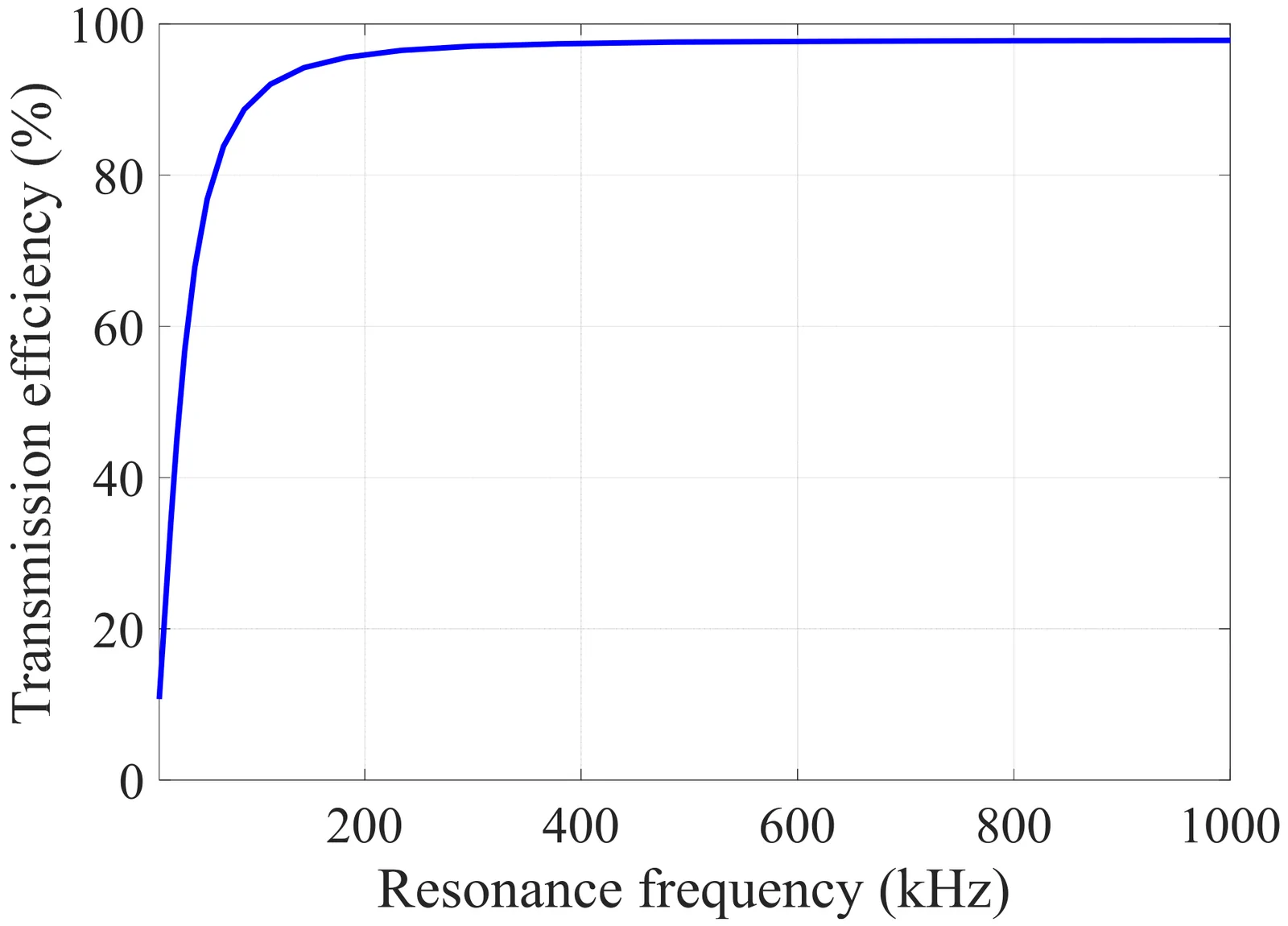
Transmission efficiency versus resonance frequency in air.

The above derivation indicates that in air, a higher resonance frequency ($f_{0}=\omega_{0}/2\pi$) is beneficial for improving the PTE, as shown in Figure 2. However, due to the good conductivity of seawater, the MCR-WPT system in a seawater environment inevitably incurs eddy current loss, which gives rise to Joule heating and consequently reduces the system efficiency. Therefore, the evaluation of system transmission efficiency in seawater should take eddy current loss into account.

### 2.2. Analysis of WPT Systems in Water

#### 2.2.1. Magnetic Flux Density Analysis

**Figure 3:**
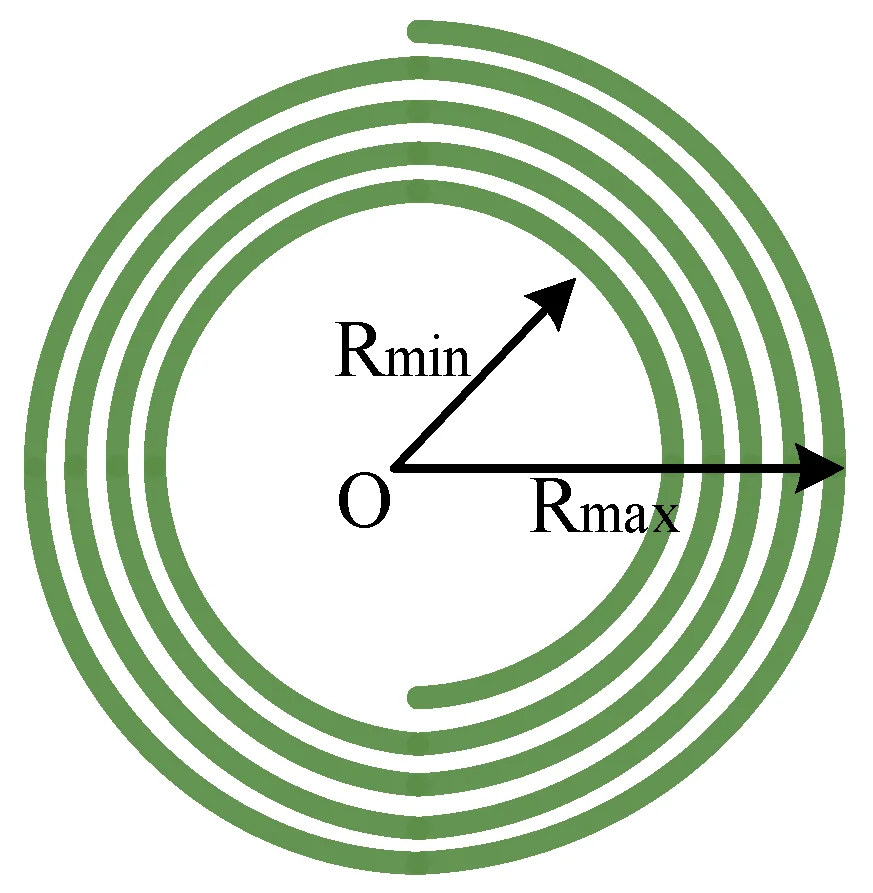
Planar circular spiral coil structure.

**Figure 4:**
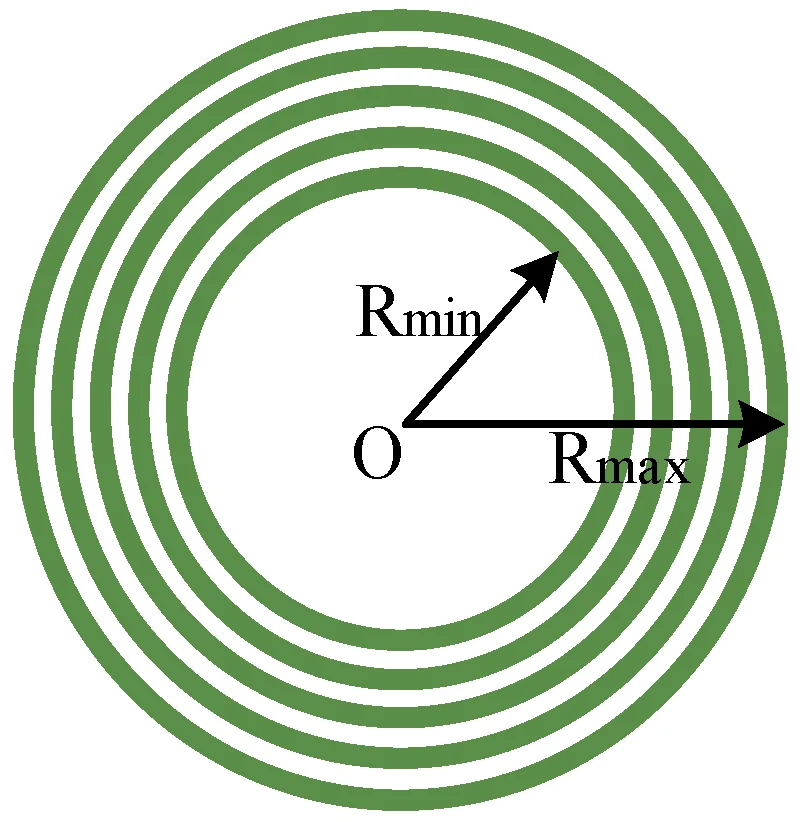
Concentric circular coil structure.

The coil adopted in this study features a planar circular spiral structure, as illustrated in Figure 3. The wire is wound into a planar spiral with a wire radius of *r*, a turn-to-turn spacing (pitch) of *w*, a maximum radius of $R_{\max}$, a minimum radius of $R_{\min}$, and a total number of turns *N*. For theoretical analysis, the coil can be approximated as *N* concentric circular coils with different radii and a uniform turn-to-turn spacing of *w*, as depicted in Figure 4.

**Figure 5:**
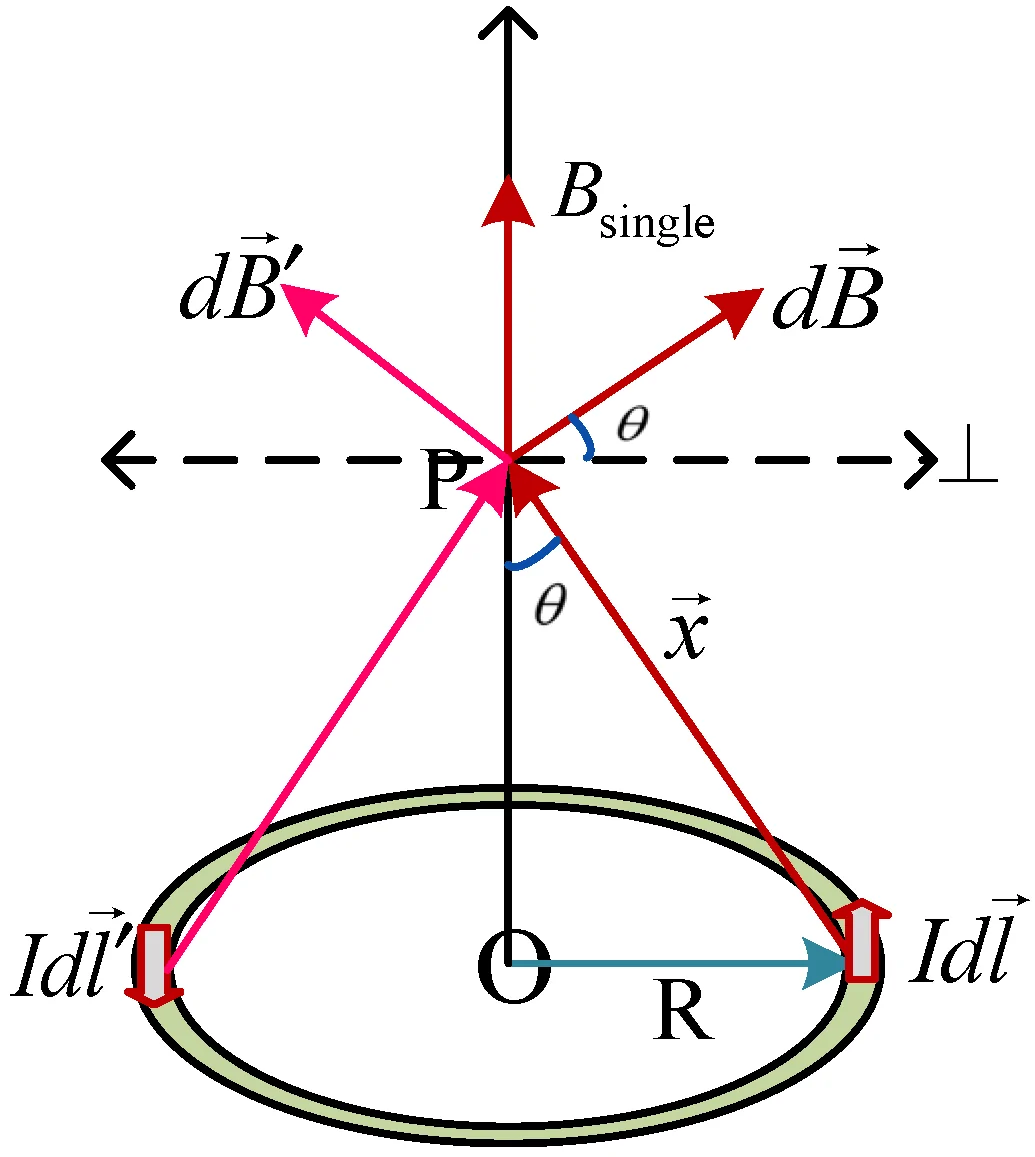
$B_{\mathrm{single}}$ generated by a single-turn coil.

The schematic diagram of the magnetic flux density generated by a single-turn coil is illustrated in Figure 5. According to the Biot–Savart law, when carrying a current $\dot{I}$, a single-turn coil of radius *R* produces a magnetic flux density at point P(0, Z) expressed as follows:

(6) $$B_{\mathrm{single}}=\frac{\mu_{0}\dot{I}R}{4\pi x^{3}}\int_{0}^{2\pi R}dl=\frac{\mu_{0}\dot{I}}{2}\frac{R^{2}}{x^{3}}=\frac{\mu_{0}\dot{I}}{2}\frac{R^{2}}{{\left(Z^{2}+R^{2}\right)}^{\frac{3}{2}}}$$

**Figure 6:**
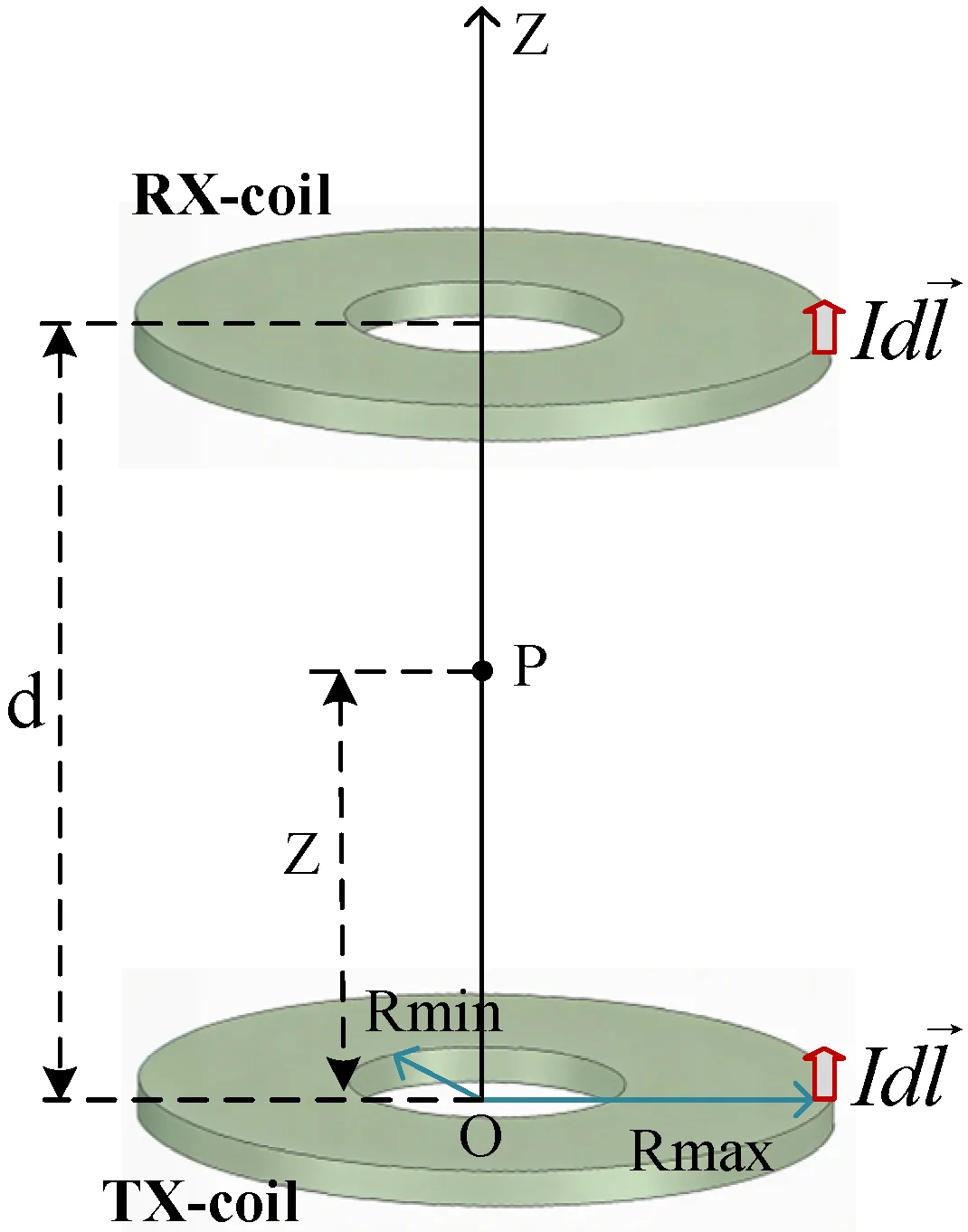
Equivalent model of the coil as a hollow disk.

**Figure 7:**
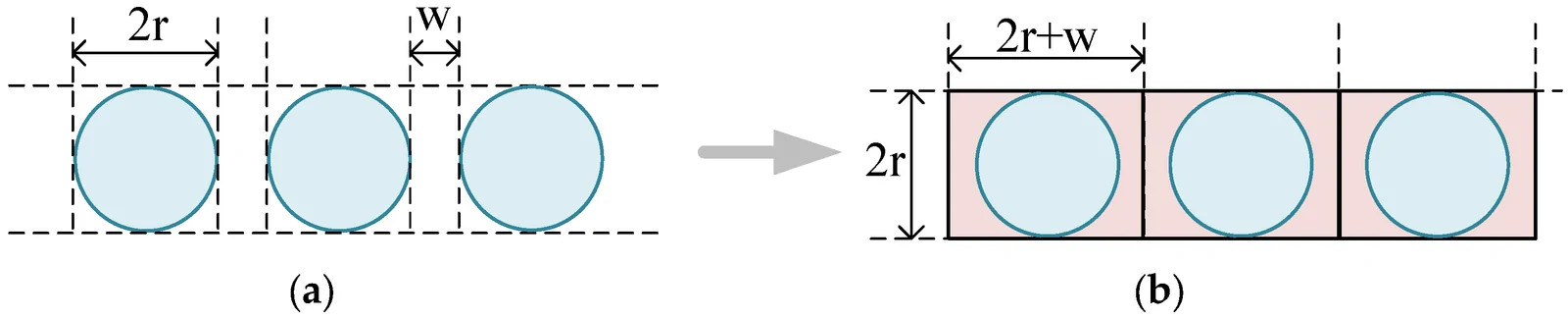
Tangential cross-sections along the Z-axis (taking a 3-turn coil as an example): (**a**) before equivalence; (**b**) after equivalence.

When the pitch is sufficiently small, the coil can be approximated as a hollow disk for calculation purposes, where *d* denotes the distance between the two coils as illustrated in Figure 6. The tangential cross-sections along the Z-axis before and after the coil is approximated as a hollow disk are presented in Figure 7.

The current magnitude in the coil remains the same before and after equivalence, $\dot{I}=\int \dot{J}dS$; therefore, the equivalence is essentially applied to the current density. At resonance, the equivalent current densities at the transmitting side and the receiving side are as follows:

(7) $${\dot{J}}_{1}^{\prime }=\frac{{\dot{I}}_{1}}{2r\left(2r+w\right)},\;{\dot{J}}_{2}^{\prime }=\frac{{\dot{I}}_{2}}{2r\left(2r+w\right)}$$

From Figure 7b and $\dot{I}=\int \dot{J^{\prime }}dS$, it can be derived that

(8) $$d\dot{I}=\dot{J^{\prime }}dS=\dot{J^{\prime }}2rdR$$

As shown in Figure 6, for a disk of radius *R*, carrying a current $\dot{I}$, the magnetic flux density *B* is obtained by integrating (6)

(9) $$B=\int_{R\min}^{R\max}\frac{\mu_{0}R^{2}}{2{\left(Z^{2}+R^{2}\right)}^{\frac{3}{2}}}d\dot{I}$$

Define $D=\ln\frac{R_{\max}+\sqrt{d^{2}+R_{\max}^{2}}}{R_{\min}+\sqrt{d^{2}+R_{\min}^{2}}}-\frac{R_{\max}}{\sqrt{d^{2}+R_{\max}^{2}}}+\frac{R_{\min}}{\sqrt{d^{2}+R_{\min}^{2}}}$, then the total magnetic flux density at the origin (Z = 0) is as follows:

(10) $$\begin{array}{l} {\dot{B}}_{0}=\mu_{0}{\dot{J}}_{1}^{\prime }r\ln\frac{R_{\max}}{R_{\min}}+\mu_{0}{\dot{J}}_{2}^{\prime }rD\\ =\frac{\mu_{0}{\dot{V}}_{s}}{2\left(2r+w\right)\left[R_{1}(R_{2}+R_{L})+\omega_{0}^{2}M_{12}^{2}\right]}\left[(R_{2}+R_{L})\ln\frac{R_{\max}}{R_{\min}}-j\omega_{0}M_{12}D\right] \end{array}$$

#### 2.2.2. Analysis of Eddy Current Loss in Water

The conductivity of seawater typically ranges from 3.1 S/m to 5.2 S/m [35], which is substantially higher than that in air. Consequently, the electromagnetic behavior in a seawater environment is more complex. Other factors, including relative permittivity, permeability, temperature, salinity, and pressure effects, may also influence the MCR-WPT system. The values and detailed descriptions of these physical quantities are listed in Table 1.

**Figure 8:**
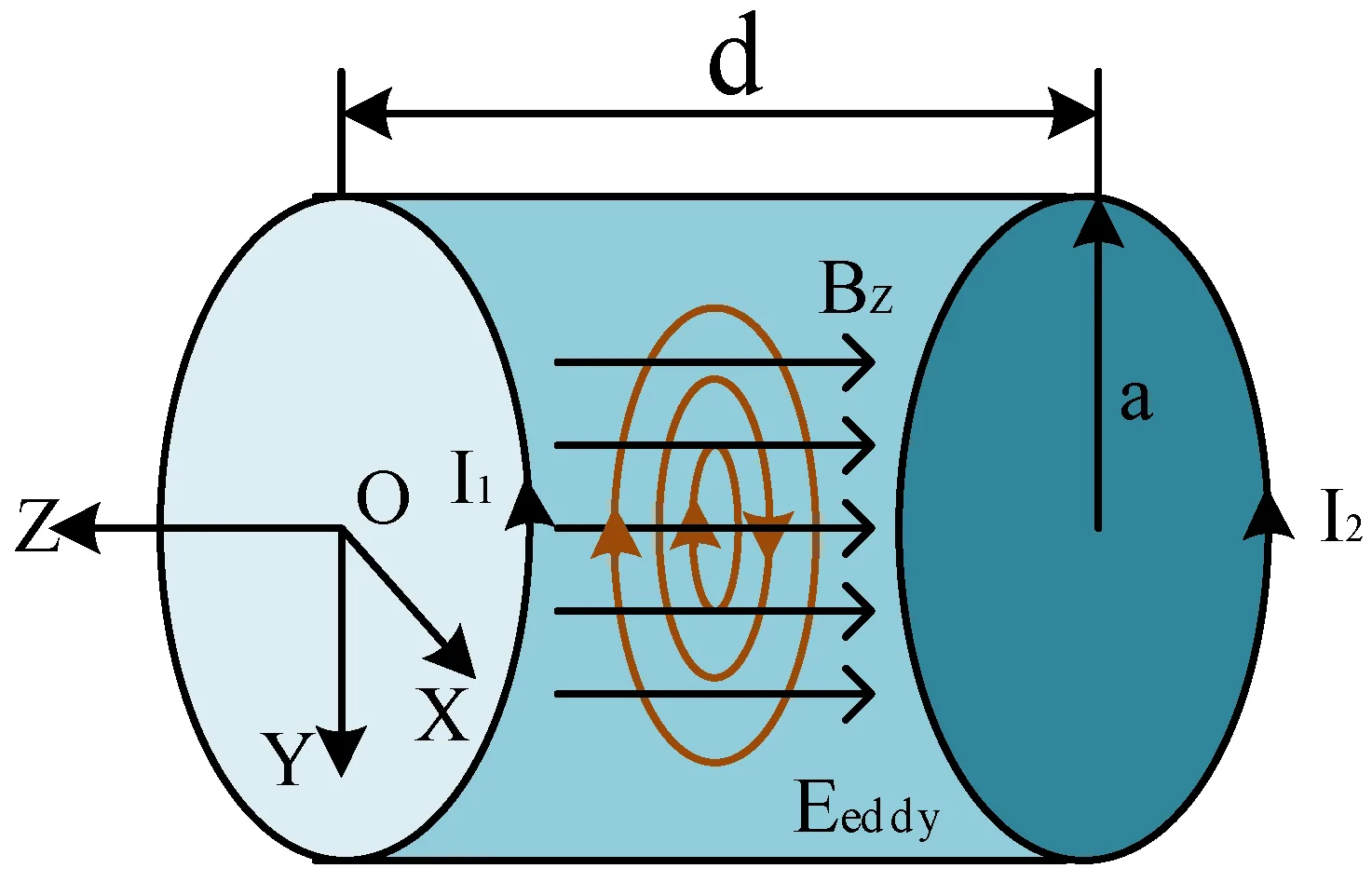
Induced eddy current phenomenon in the underwater MCR-WPT system.

Therefore, the above physical quantities are not the primary focus of this study. When the coil is immersed in seawater, the simplified schematic is presented in Figure 8.

(11) $$P_{\mathrm{eddy}}={\int_{V}\sigma \left|{\dot{E}}_{y}\right|}^{2}dV$$

Let $\beta =\sqrt{\frac{\omega_{0}\mu \sigma }{2}}$ and k=(1+j)β. The general solution for the electric field intensity in the y-direction can be expressed in hyperbolic cosine form as ${\dot{E}}_{y}=-\frac{{\dot{B}}_{0}k}{\mu \sigma }\sinh(1+j)\beta x$. The equivalent radius a of the coil is defined as $a=\frac{R_{\max}+R_{\min}}{2}$. The eddy current loss generated in the homogeneous water medium between the coils can then be expressed as follows:

(12) $$\begin{array}{l} P_{eddy}=\int_{V}\sigma {\left|{\dot{E}}_{y}\right|}^{2}dV=\int_{V}\sigma {\left|\frac{{\dot{B}}_{0}k}{\mu \sigma }\sinh(1+j)\beta x\right|}^{2}dV\\ =\int_{V}\sigma {\left|\frac{{\dot{B}}_{0}k}{\mu \sigma }\right|}^{2}{\left|\frac{e^{(1+j)\beta x}-e^{-(1+j)\beta x}}{2}\right|}^{2}dV\\ =\frac{\omega_{0}\pi d}{\mu }{\left|{\dot{B}}_{0}\right|}^{2}\int_{0}^{a}\left(\cosh2\beta x-\cos2\beta x\right)xdx\\ =\frac{\omega_{0}\pi d}{\mu }{\left|{\dot{B}}_{0}\right|}^{2}\left(\frac{a(\sinh2\beta a-\sin2\beta a)}{2\beta }-\frac{\cosh2\beta a+\cos2\beta a}{4\beta^{2}}+\frac{1}{2\beta^{2}}\right) \end{array}$$

where

V—the cylindrical seawater volume between the two coils;

μ—permeability;

σ—conductivity;

$E_{y}$—Y-directed electric field intensity.

The hyperbolic function sinh2βa and cosh2βa, the trigonometric function sin2βa and cos2βa in the above equation are expanded using Taylor series. When 2βa≤1, the value of βa approaches zero as the order increases, and its effect on the calculation result becomes negligible. Therefore, neglecting the fifth- and higher-order terms and substituting β yields:

(13) $$P_{eddy}\approx \frac{\omega_{0}^{2}\pi d\sigma a^{4}}{2}{\left|{\dot{B}}_{0}\right|}^{2}$$

${\dot{B}}_{0}$ is obtained by solving (10). According to (13), $P_{\mathrm{eddy}}$ is influenced by factors such as conductivity, resonance frequency, and the distance between the coils, which in turn affects the system efficiency. Referring to the system parameters listed in Table 2, numerical analysis is carried out using MATLAB (R2026a).

**Figure 9:**
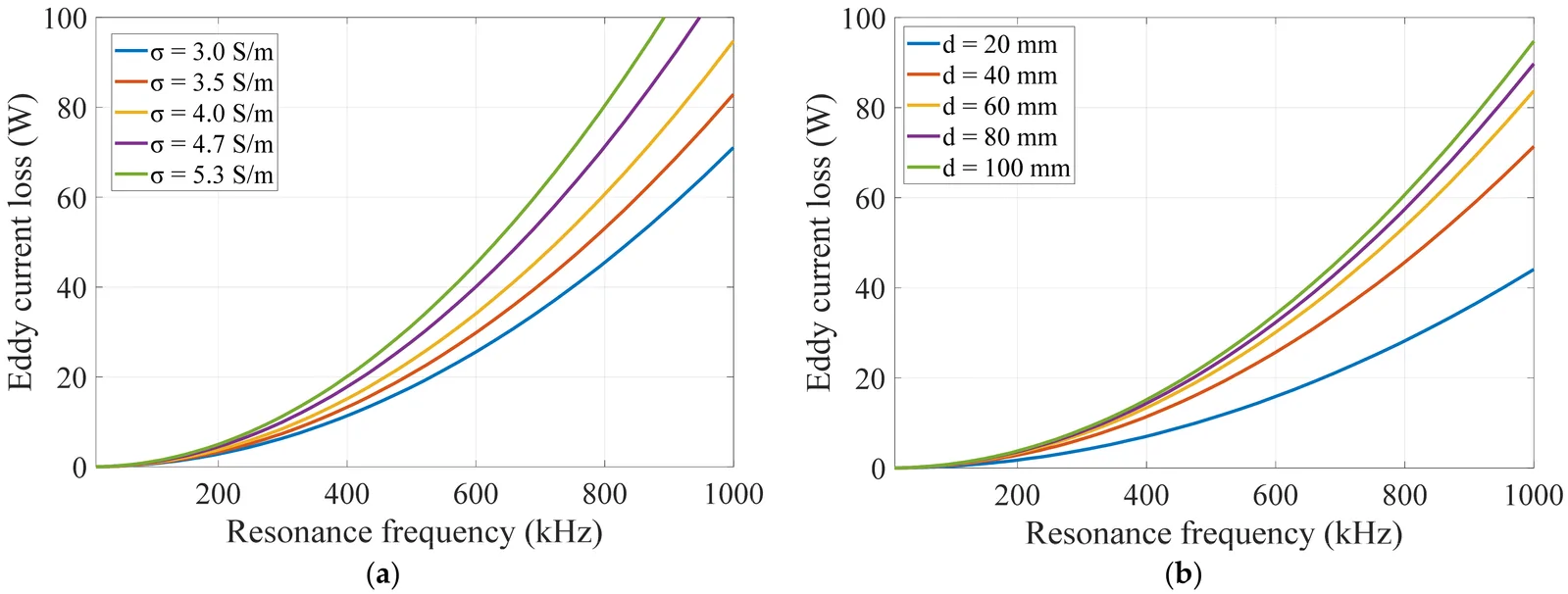
$P_{\mathrm{eddy}}$ versus $f_{0}$: (**a**) under different σ; (**b**) at different d.

Figure 9a shows the eddy current loss $P_{\mathrm{eddy}}$ versus resonance frequency $f_{0}$. The results are obtained in underwater environments with different conductivities. The transmission distance is 100 mm, and both coils are excited by current sources. It is seen that $P_{\mathrm{eddy}}$ increases with $f_{0}$ under all conductivity conditions, and the growth is faster at higher $f_{0}$. Also, a larger σ gives a larger $P_{\mathrm{eddy}}$. Figure 9b shows $P_{\mathrm{eddy}}$ versus $f_{0}$, at a conductivity of 4 S/m, for different transmission distances. It is seen that $P_{\mathrm{eddy}}$ increases with $f_{0}$ for all distances, and the growth is faster at higher $f_{0}$ Also, a larger distance leads to a larger $P_{\mathrm{eddy}}$.

The above findings are consistent with most experimental results reported in the literature. In air, the system efficiency increases with $f_{0}$. Therefore, an optimal resonance frequency exists that maximizes the efficiency, and excessively high resonant frequencies are not suitable for seawater environments.

#### 2.2.3. Analysis of MCR-WPT Systems in Water

**Figure 10:**
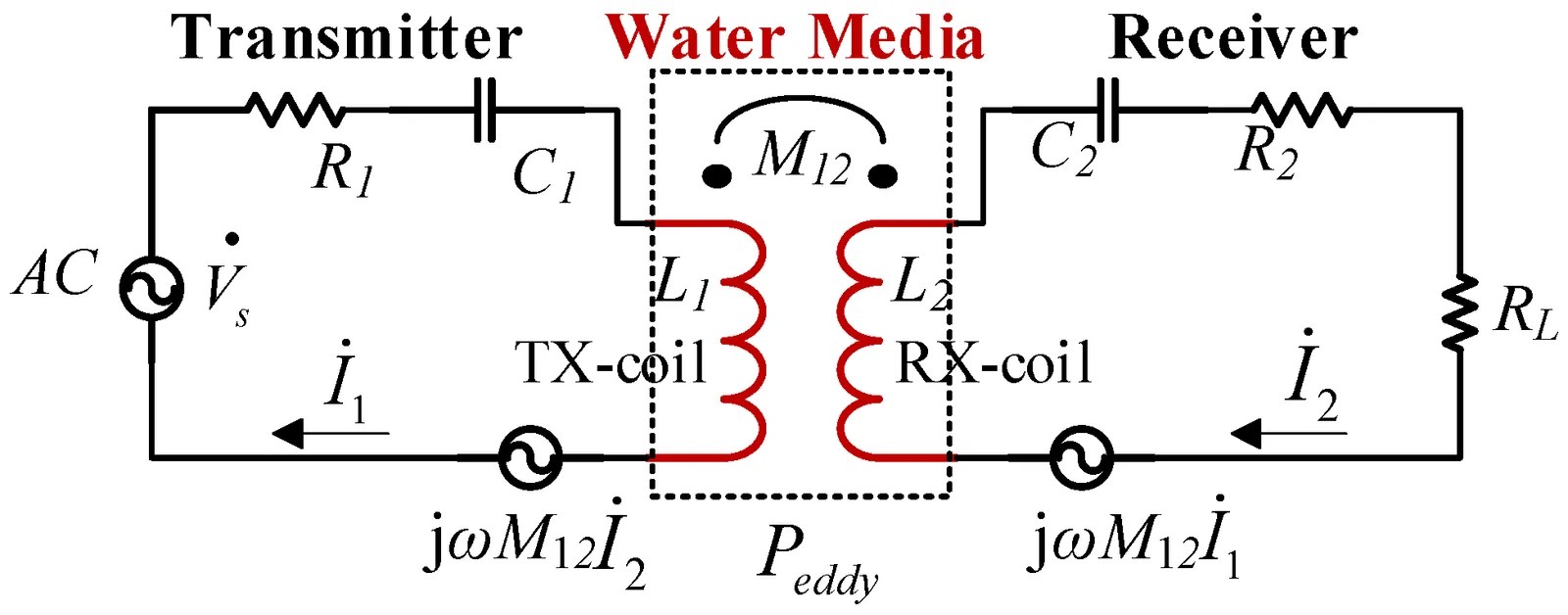
Equivalent circuit model (ECM) of the MCR-WPT system in water.

With reference to the ECM in Figure 10, and based on the system efficiency in air (see (4)), the eddy current loss in water $P_{\mathrm{eddy}}$ from (13) is incorporated. The transmission efficiency in the seawater environment is calculated using $\eta_{water}=\frac{P_{R_{L}}}{P_{in}+P_{eddy}}$. In this study, $P_{RL}$ denotes the load power, while $P_{in}$ denotes the active power at the transmitting side. The latter is measured at the transmitting-side feeding port. The input power measured at this port includes the copper loss of the transmitting coil, whereas the eddy current loss in seawater occurs along the transmission path between the transmitting and receiving coils. Therefore, the denominator is the sum of the active power at the transmitting side and the power associated with eddy current loss, which does not result in double counting.

By defining $\xi =\frac{\pi d\sigma a^{4}}{2}$, the efficiency (PTE) of the MCR-WPT system in homogeneous water under resonance can be expressed as follows:

(14) ηwater=PRLPin+Peddy=I˙22RLReV˙sI˙1+ξω02B˙02=ω02M122RLR2+RLR1(R2+RL)+ω02M122+μ02ξω0242r+w2(R2+RL)2lnRmaxRmin2+ω02M122D2

Taking the first-order partial derivative of $\eta_{water}$ with respect to the resonant angular frequency $\omega_{0}$ in (14), and setting $\partial \eta_{water}/\partial_{\omega_{0}}=0$, yields:

(15) $$\omega_{0-\mathrm{opti}}=\frac{1}{a}\sqrt{\frac{2\left(2r+w\right)(R_{2}+R_{L})}{\mu_{0}M_{12}D}\sqrt{\frac{2R_{1}}{\pi d\sigma }}}$$

Equation (15) indicates that there is an optimal resonance frequency in seawater for maximizing the PTE of the MCR-WPT system. The optimal angular frequency $\omega_{0-\mathrm{opti}}$ decreases as conductivity increases, and is also affected by coil parameters and transmission distance. It should be noted that, in the strong-coupling frequency-splitting region, if the system selects one of the two peaks as the resonance frequency, the applicability of this expression needs to be re-evaluated or the expression should be modified accordingly.

**Figure 11:**
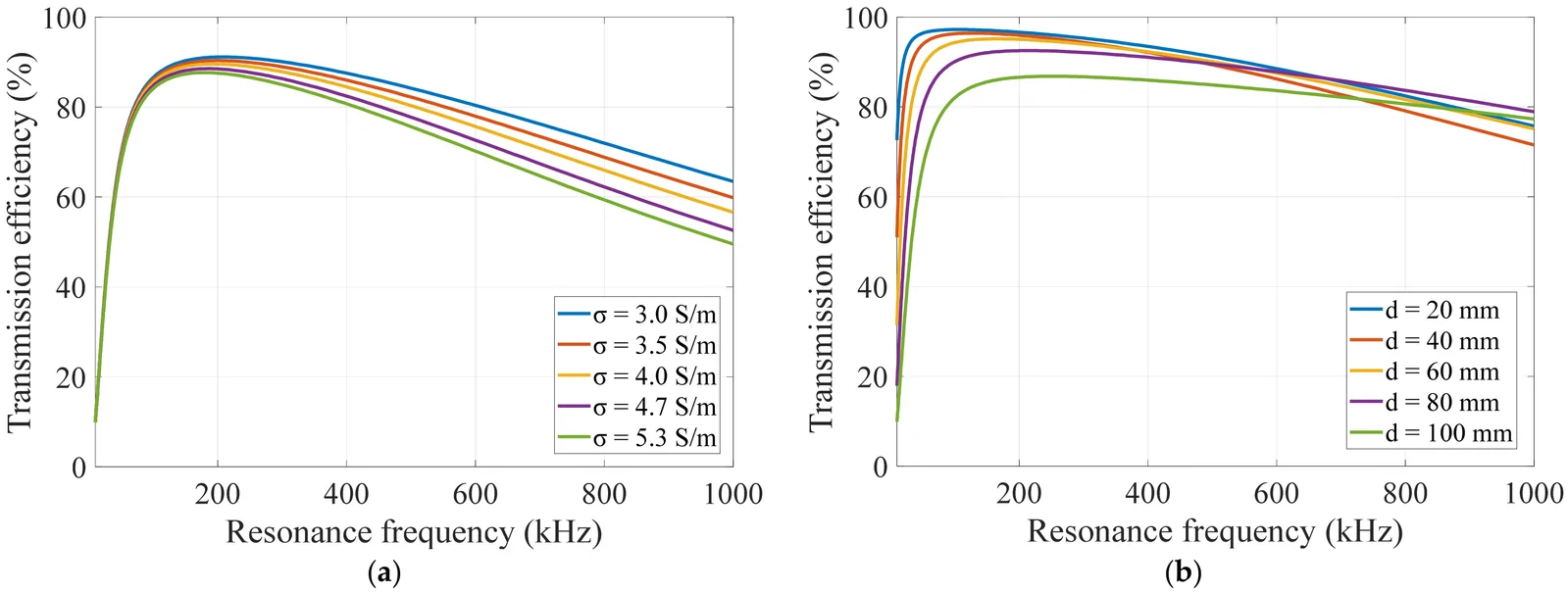
$\eta_{water}$ versus $f_{0}$: (**a**) under different σ; (**b**) at different d.

Figure 11a shows the variation in transmission efficiency with resonance frequency $f_{0}$ under different conductivity levels. It can be observed that $\eta_{water}$ first increases and then decreases as $f_{0}$ rises. At the same frequency, higher conductivity gives lower efficiency. Also, the optimal resonance frequency $f_{0-\mathrm{opti}}$ ($f_{0-opti}=\omega_{0-opti}/2\pi$) becomes smaller when conductivity increases. Figure 11b presents $\eta_{water}$ versus $f_{0}$ at different transmission distances. It is seen that the efficiency decreases with distance when the resonance frequency is below 300 kHz.

It should be noted that the above model is derived using idealized assumptions, such as a homogeneous medium and $B_{0}$. The purpose is to establish an analytical estimation model and derive explicit expressions for evaluating eddy current loss and transmission efficiency. The model also describes their relationships with key parameters, including resonance frequency, conductivity, and transmission distance. The predicted trends are qualitatively consistent with the subsequent simulation and experimental results. This analysis serves as a conceptual framework for supporting and interpreting the subsequent simulations and experiments, rather than providing a precise analytical description. At present, comprehensive finite element analysis (FEA), including the off-axis magnetic field distribution and volume integration of seawater loss, remains the reference method for accurate quantitative prediction of loss.

## 3. Underwater Simulation Analysis

To investigate the effect of resonance frequency on system performance in underwater environments, finite element analysis (FEA) is employed to simulate the WPT system in seawater. The effects of resonance frequency and conductivity on eddy current loss and transmission efficiency are examined. Since Maxwell is mainly used for electromagnetic field analysis and does not provide circuit simulation capabilities, only the coupling field between the two coils is simulated in this study. The load and compensation capacitors are not included in the simulation. A frequency sweep is performed to obtain the results at different resonant frequencies.

**Figure 12:**
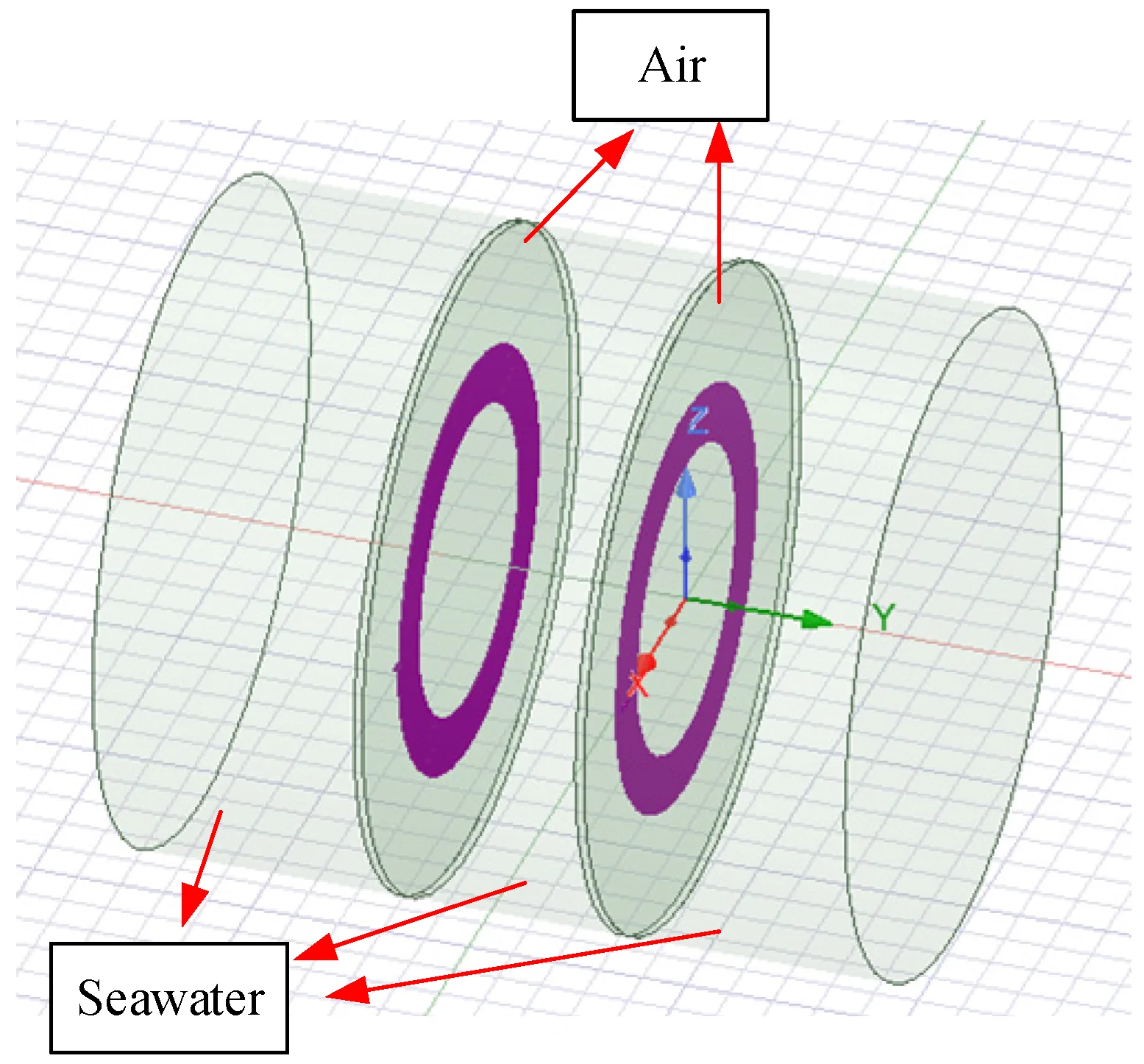
Simulation model of the WPT system in seawater.

The WPT system model in seawater is illustrated in Figure 12. The simulation adopts a planar circular spiral coil as the coupling coil, with parameters given in Table 1. A thin air layer is placed around the coil to represent the waterproof coating, while the remaining domain is assigned appropriate conductivity and relative permittivity to model the seawater environment. The detailed simulation parameters are listed in Table 3.

### 3.1. Effect of Conductivity on Magnetic Field Propagation

**Figure 13:**
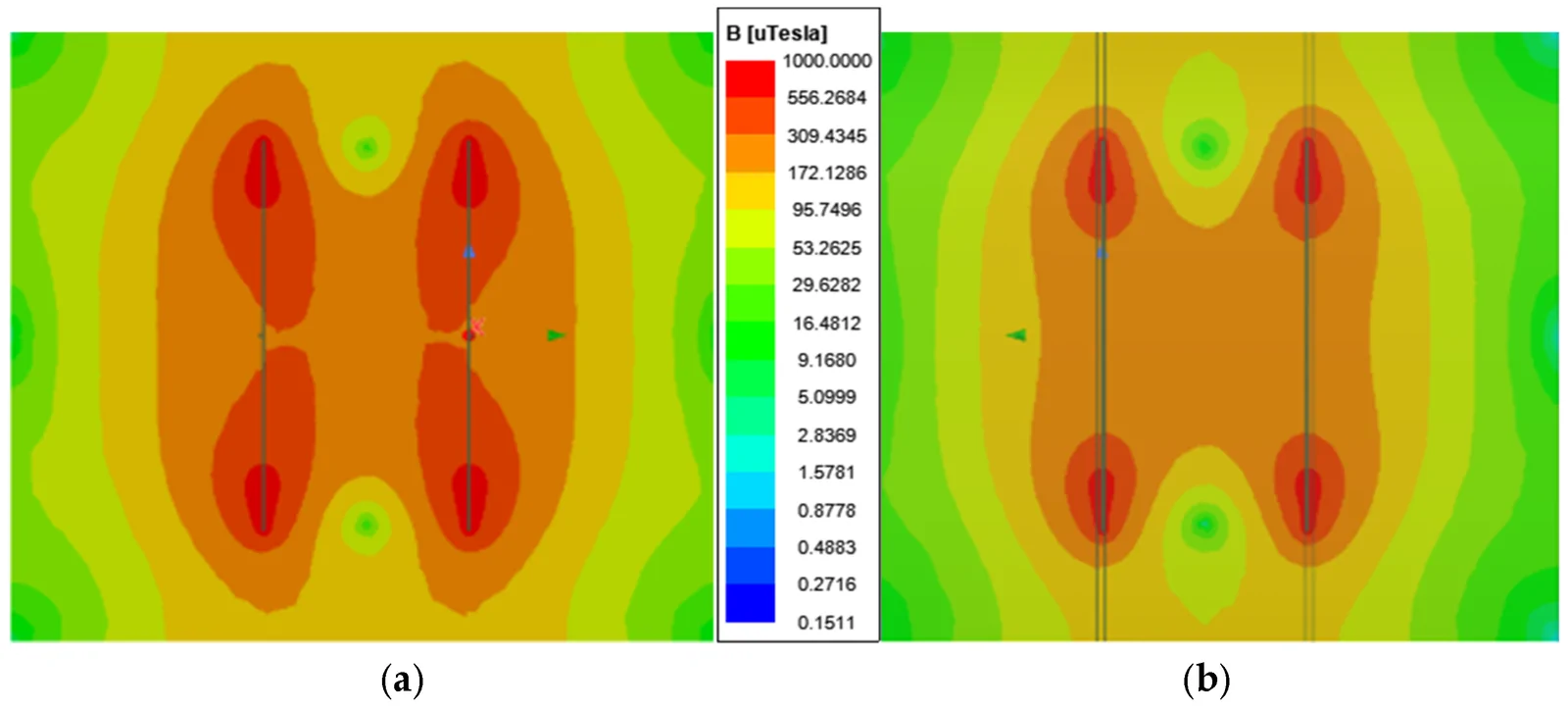
Distribution of magnetic flux density in underwater environments: (**a**) in freshwater with a conductivity of 0.05 S/m; (**b**) in seawater with a conductivity of 4 S/m.

Figure 13 presents the distribution of magnetic flux density in freshwater (0.05 S/m) and seawater (4 S/m). The simulation results show that the magnetic flux density is significantly higher in freshwater. It is mainly concentrated around the coil, and decays with increasing distance from the coil. This indicates that the magnetic field energy weakens gradually as the transmission distance increases.

**Figure 14:**
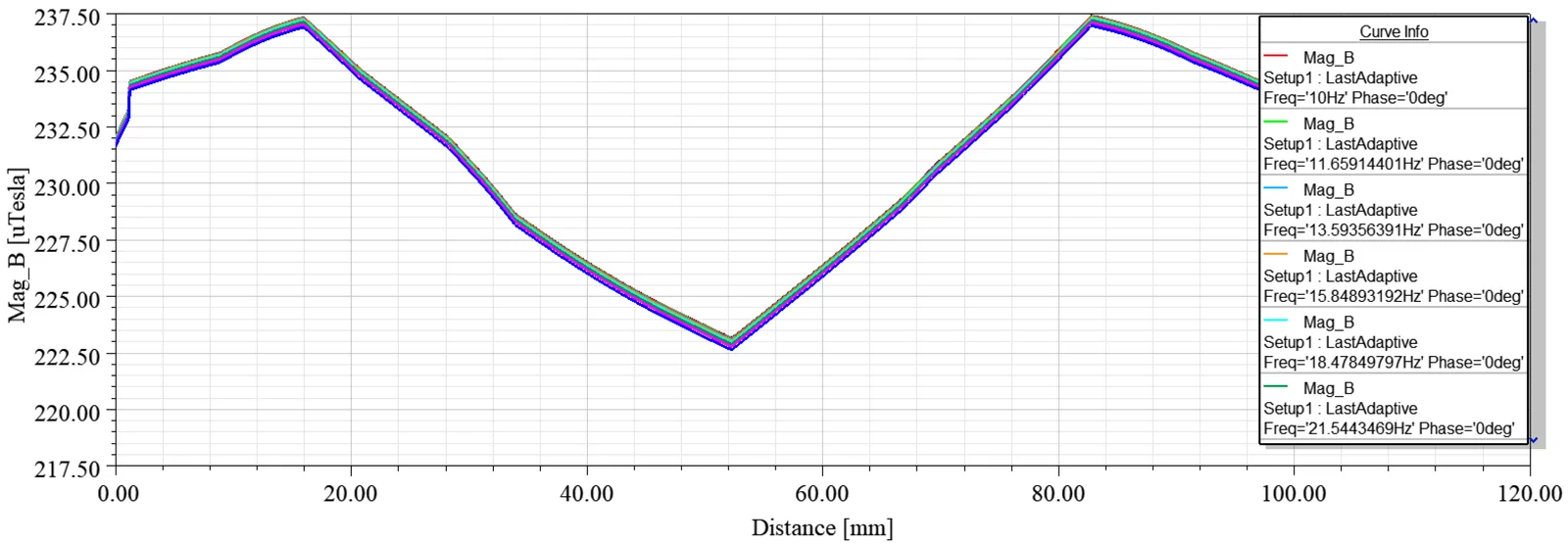
Simulation Results of Magnetic Flux Density Along the Axial Direction.

To quantify the error introduced by the proposed equivalent model, a relative error analysis (Relative Error=Theoretical Value−Simulated ValueSimulated Value) was performed on the $B_{0}$ obtained from theoretical calculations and simulation analysis under the same conditions. A total of 77 frequency points were selected over the resonance frequency range from 1 Hz to 1 MHz for point-by-point comparison. The relative error remained stable at approximately 22.8% and was nearly constant over the entire frequency range. This discrepancy arises because the magnetic flux density is not constant along the axial direction. Instead, it exhibits an approximately sinusoidal distribution with increasing axial distance, with the center of the transmitting coil taken as the origin, as shown in Figure 14. Since this error remains nearly constant over the frequency domain, it does not substantially affect the determination of the resonance frequency. Therefore, within the framework of this study, the $B_{0}$ approximation is acceptable and effective, and the systematic deviation introduced by this approximation does not affect the reliability of the main conclusions.

**Figure 15:**
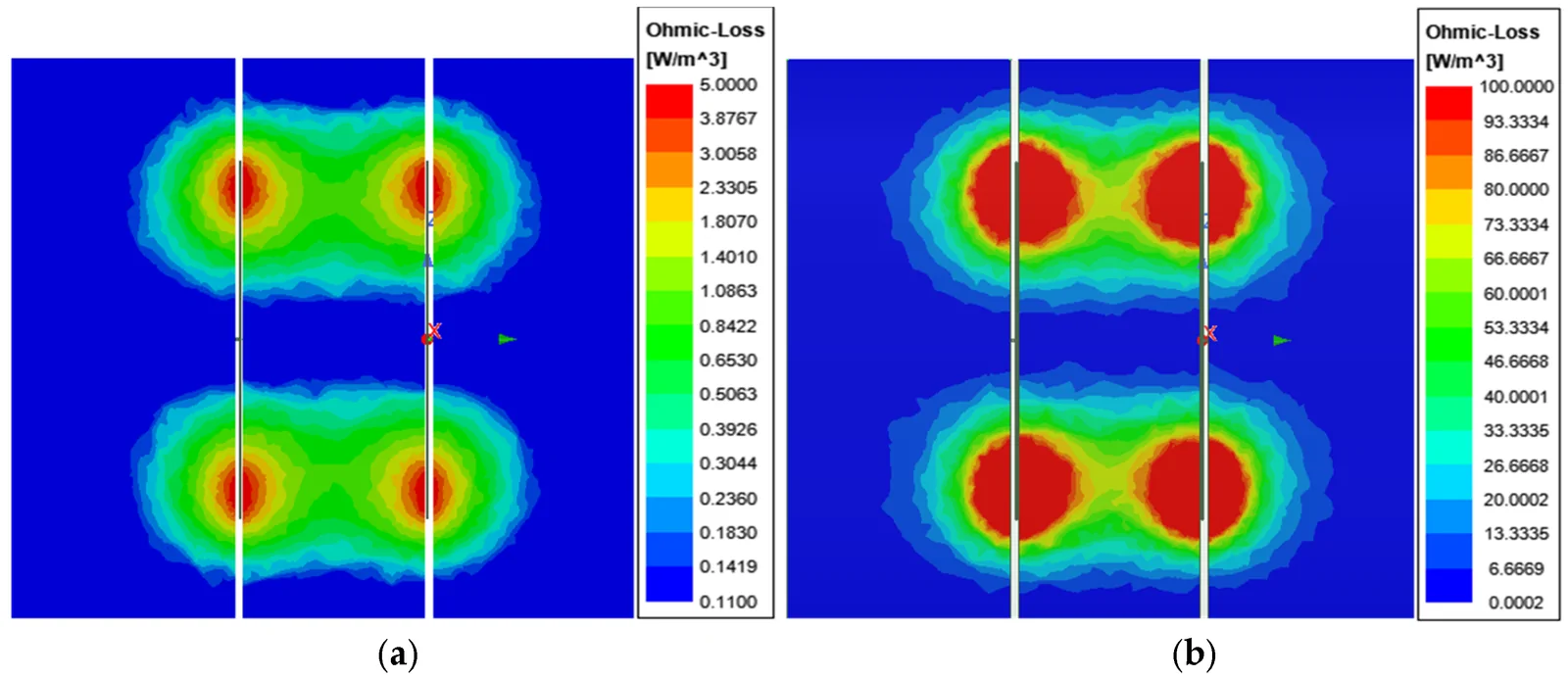
Distribution of eddy current loss in underwater environments: (**a**) in freshwater with a conductivity of 0.05 S/m; (**b**) in seawater with a conductivity of 4 S/m.

Figure 15 presents the simulated eddy current loss distribution in freshwater and seawater environments. The distribution pattern is generally consistent with that of the magnetic flux density. The eddy current loss in freshwater is significantly lower than that in seawater, and is mainly concentrated near the coil. It decays gradually with increasing distance from the coil. This is because the eddy current loss induced in highly conductive seawater exacerbates the attenuation of magnetic field energy, thereby reducing the magnetic flux density. This indicates that high conductivity significantly affects magnetic field propagation.

### 3.2. Resonance Frequency Characteristics of WPT Systems Under High Conductivity

**Figure 16:**
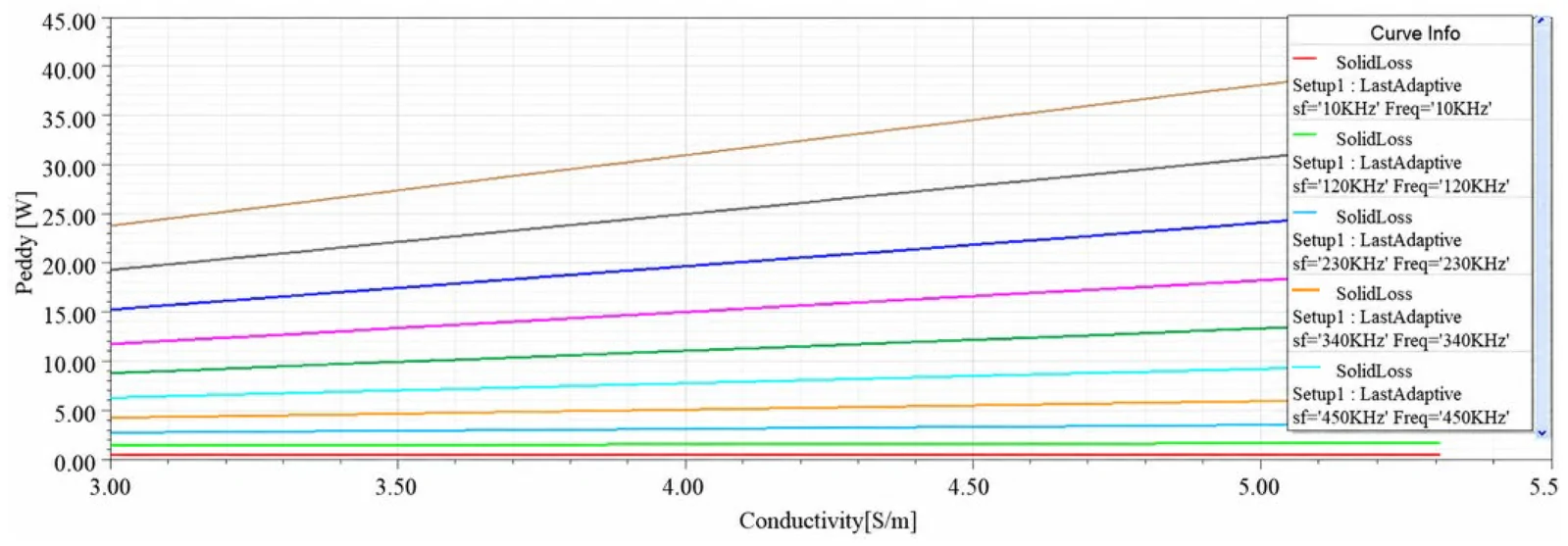
Simulation results of $P_{\mathrm{eddy}}$ versus σ at different resonant frequencies.

Since seawater temperature varies with depth, and conductivity is closely related to temperature, the effect of different conductivity levels on $P_{\mathrm{eddy}}$ is investigated through simulation. The seawater conductivity is varied from 3 S/m to 5.3 S/m, with a relative permittivity of 81. A constant current excitation is applied, and frequency sweeping is performed in the simulation. Figure 16 presents the simulation results of $P_{\mathrm{eddy}}$ versus σ at different resonant frequencies. It is observed that at each resonance frequency, $P_{\mathrm{eddy}}$ increases with conductivity, and the trend becomes more pronounced at higher conductivity levels. At a given conductivity, $P_{\mathrm{eddy}}$ also rises with increasing resonance frequency. The effect of eddy current loss is negligible at low frequencies, but becomes increasingly significant as the frequency increases. This indicates that high resonant frequencies are unsuitable for WPT systems operating in high-conductivity seawater.

A relative error analysis (Relative Error=Theoretical Value−Simulated ValueSimulated Value) was performed on the eddy current loss obtained from the simulations and theoretical calculations. In the intermediate-frequency range, the theoretical and simulation results show good agreement, with a relative error of approximately 4.5%. At lower and higher frequencies, the error increases significantly, reaching a maximum of 53.97%. This is mainly because the relative error in magnetic flux density between the simulation and theoretical results is approximately 23%. Since eddy current loss is proportional to the square of magnetic flux density, this error is amplified. Nevertheless, the overall variation trend is consistent with the theoretical analysis, indicating that the main conclusions of this study remain reliable.

**Figure 17:**
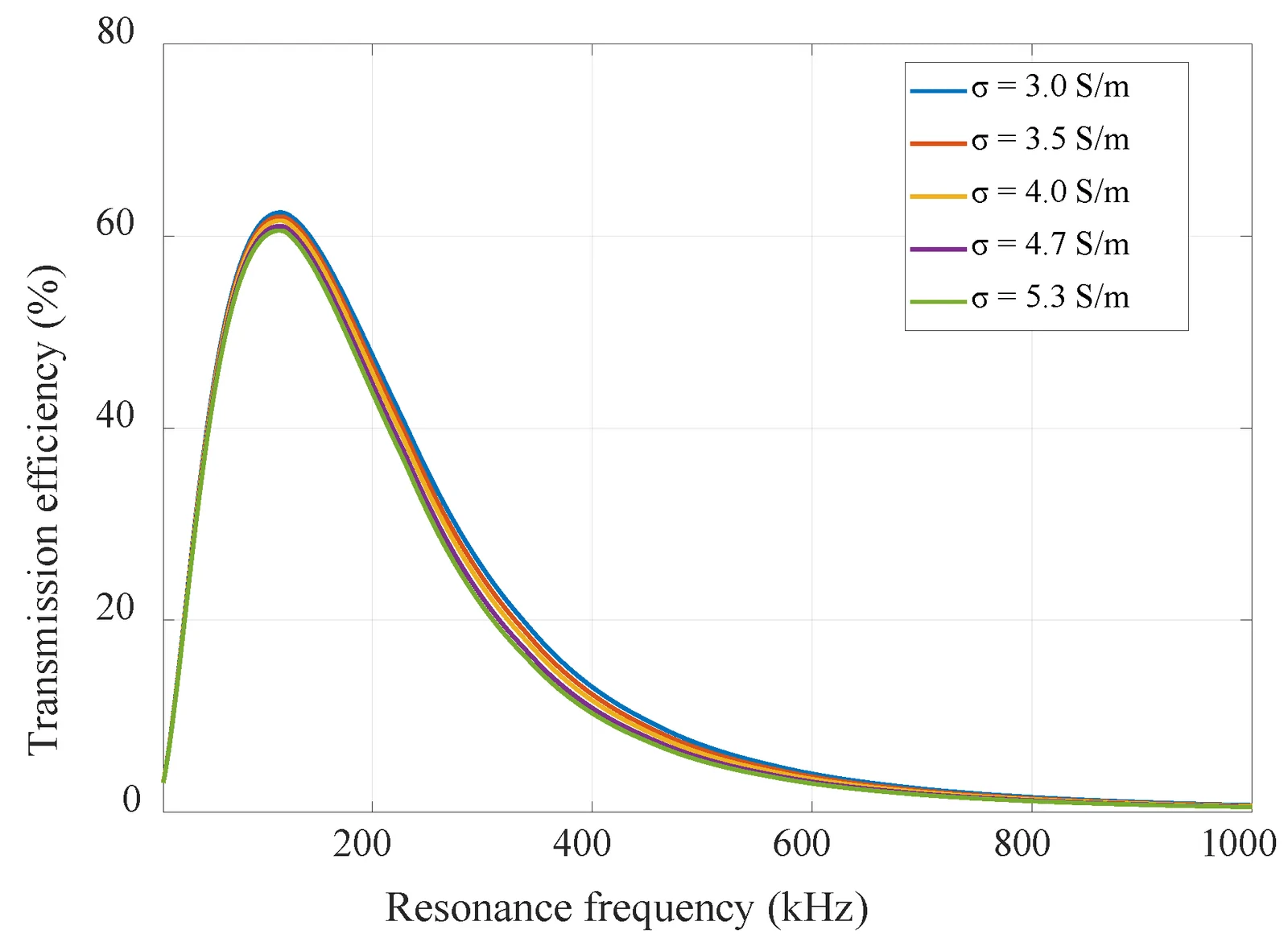
$\eta_{water}$ versus $f_{0}$ under different conductivity levels.

To evaluate the transmission efficiency in seawater, the load power $P_{RL}$ and input active power $P_{in}$ of the WPT system are first obtained. The simulated eddy current loss is then substituted into $\eta_{water}=\frac{P_{R_{L}}}{P_{in}+P_{eddy}}$ for calculation. (Note: The definitions of the power quantities used here are consistent with those described in Section 2.2.3). Figure 17 shows the transmission efficiency versus resonance frequency in seawater with different conductivity levels. It can be observed that for all five conductivity levels, $\eta_{water}$ first increases and then decreases with $f_{0}$. This indicates that an optimal resonance frequency exists for maximizing the efficiency. Additionally, at a given resonance frequency, the efficiency decreases as conductivity increases.

It should be noted that both Figure 11 and Figure 16 describe the relationship between transmission efficiency and resonance frequency, but they follow different calculation approaches. Figure 11 presents purely numerical results calculated strictly using the theoretical model of the WPT system under constant-voltage excitation. In contrast, Figure 16 presents the results obtained by substituting the simulated eddy current loss into the transmission efficiency expression. In this case, $P_{eddy}$ is obtained from the simulation, whereas $P_{out}$ and $P_{in}$ are obtained from the theoretical analysis. The main reason is that Ansys Maxwell, the finite element analysis (FEA) software used in this study, does not provide circuit simulation capabilities. The simulation therefore considers only the coupling between the two coils. This is an important source of the discrepancy between the theoretical and simulation results.

## 4. Experimental Verification of Resonance Frequency Characteristics

**Figure 18:**
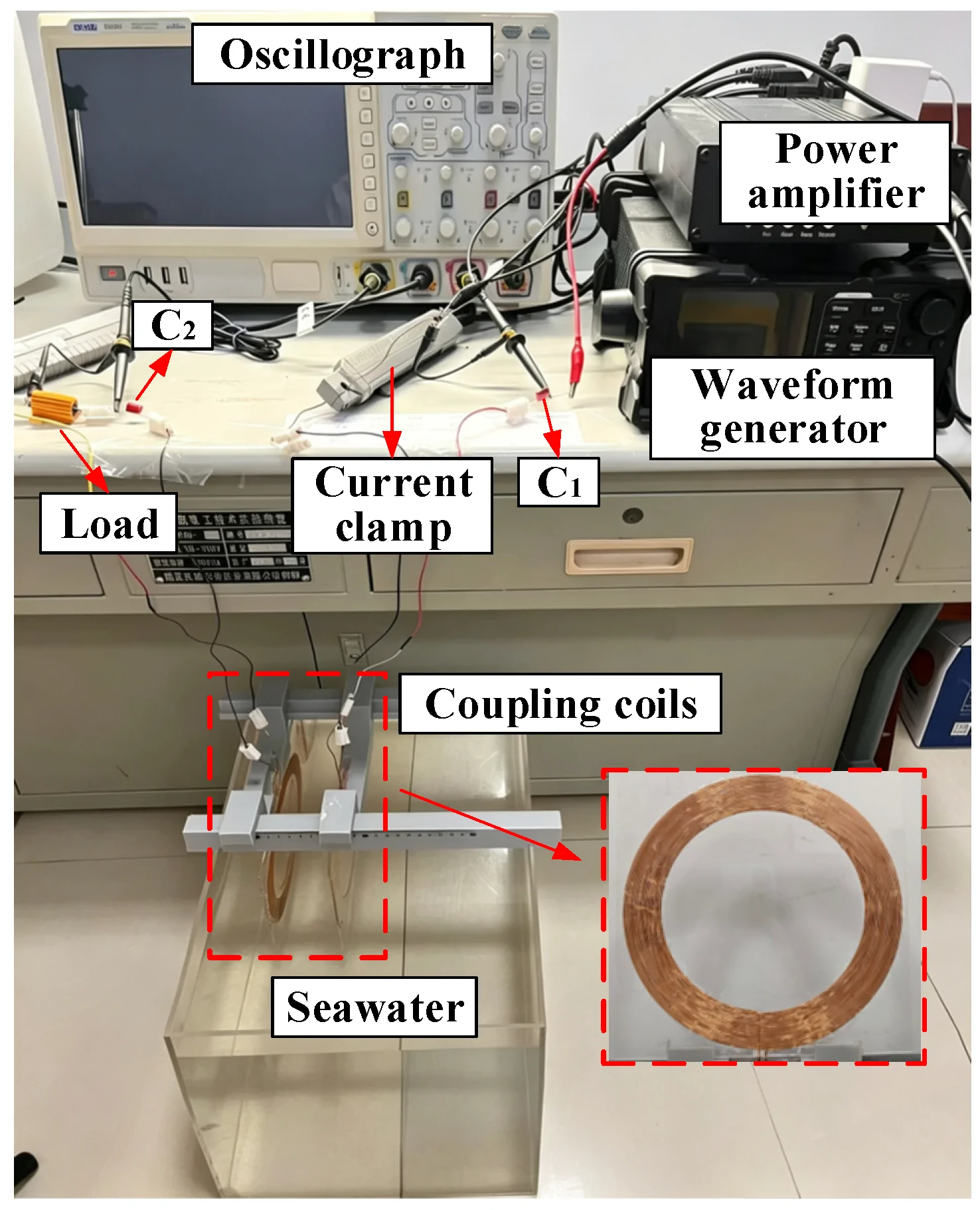
Schematic diagram of the overall experimental platform.

To further validate the theoretical and simulation results, an underwater WPT experimental platform is constructed, as illustrated in Figure 18. The transmitting-side circuit consists of a waveform generator, a power amplifier, a capacitor, and a transmitting coil. The receiving-side circuit consists of a capacitor, a load resistor, and a receiving coil. A conductivity meter is used to accurately measure the conductivity of the experimental environment. An acrylic container is filled with approximately 30 L of aqueous solution to ensure that the coils are fully submerged and sufficient space is maintained around them, thereby minimizing the influence of boundary effects on the measurements. The coil parameters are given in Table 2, and detailed information on the test instruments is shown in Table 4. During the experiments, the resonant point is identified when the phase difference between the voltage and current at the transmitting side is zero and the output voltage reaches its maximum. The voltage and current at the transmitting side and across the load are measured using an oscilloscope and a current probe to calculate the transmission efficiency. Multiple measurements are conducted at each data point, and their average is taken as the final experimental result. The experimental data are fitted using a second-order polynomial based on the least-squares method.

### 4.1. Experimental Verification in Air

**Figure 19:**
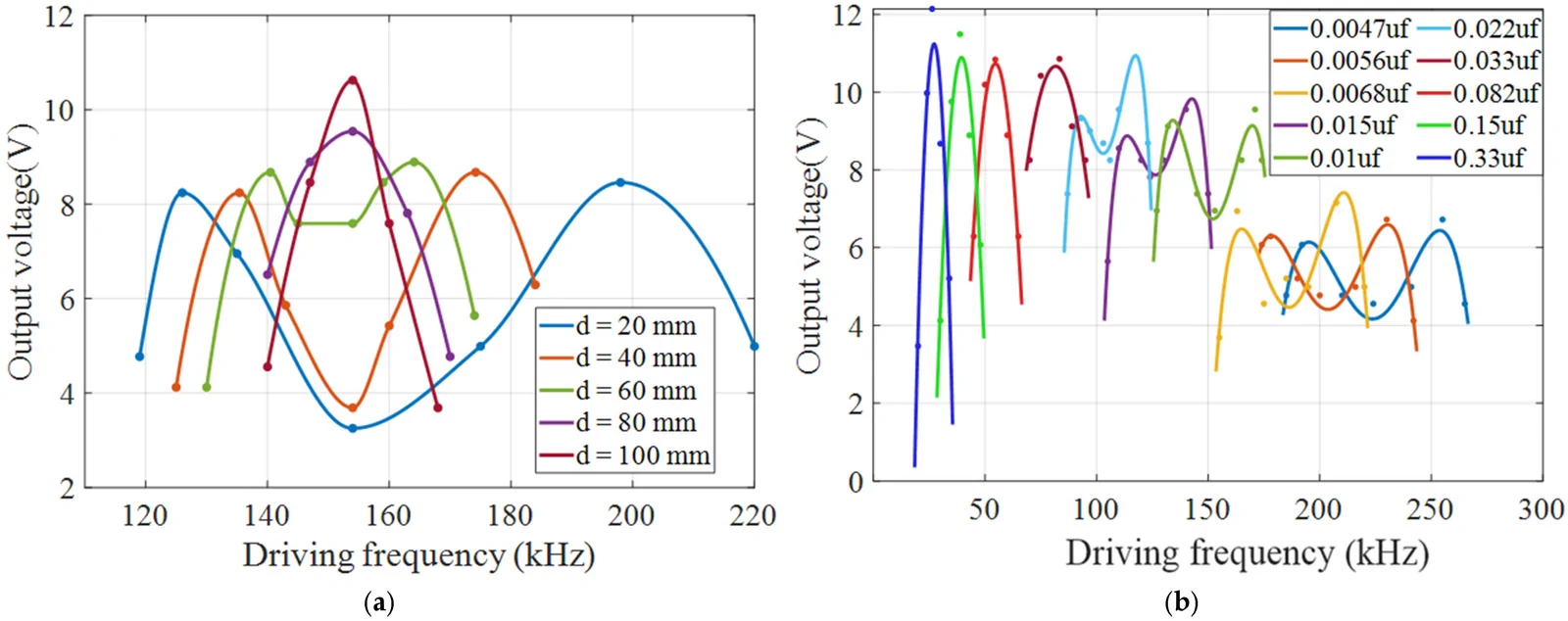
Experimental results of frequency splitting in air: (**a**) with fixed capacitance (i.e., resonance frequency); (**b**) with fixed transmission distance.

Experimental verification is first carried out in air. When the transmission distance between the coils is too small, the output voltage tends to exhibit frequency splitting. The corresponding results are shown in Figure 19. The results indicate that, for a fixed capacitance, frequency splitting begins when the transmission distance is reduced to 60 mm, and becomes more pronounced as the distance decreases further. Similarly, at a fixed transmission distance, frequency splitting starts at a capacitance of 0.022 μF, and intensifies as the capacitance decreases.

**Figure 20:**
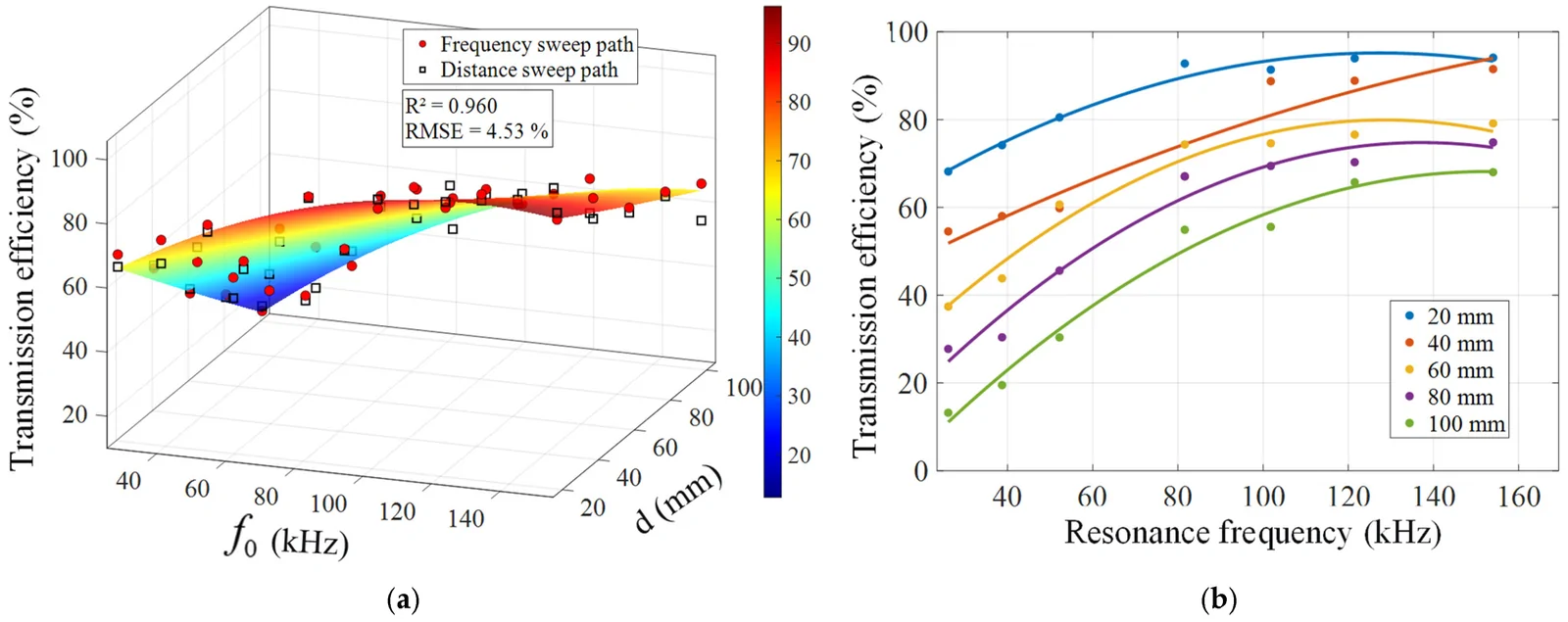
Experimental results of resonance frequency characteristics in air: (**a**) three-dimensional fitted surface; (**b**) efficiency–frequency cross-sections.

Figure 20 shows the experimental results of resonance frequency characteristics in air. The results show that the transmission efficiency in air decreases with increasing transmission distance. At a given capacitance, it increases with resonance frequency and then saturates after reaching a certain value. The three-dimensional fitting results show that the coefficient of determination $R^{2}$ reaches 0.96, indicating that the fitted surface can effectively reflect the overall variation pattern of the experimental data. The RMSE is 4.53 percentage points, indicating a relatively small overall deviation between the fitted values and the experimental data. Overall, the fitting adequately represents the general trend of the experimental results with changes in the relevant parameters.

It should be noted that the effect of transmission distance on resonance frequency was not considered in the experiments. Instead, the resonance frequency at a transmission distance of 100 mm, where frequency splitting did not occur, was used as the resonance frequency for experiments at other transmission distances under the same conductivity. This approach avoids the difficulty of identifying the true resonant point when two peaks appear during the experiments and does not affect the core research findings of this study.

### 4.2. Underwater Experimental Verification

**Figure 21:**
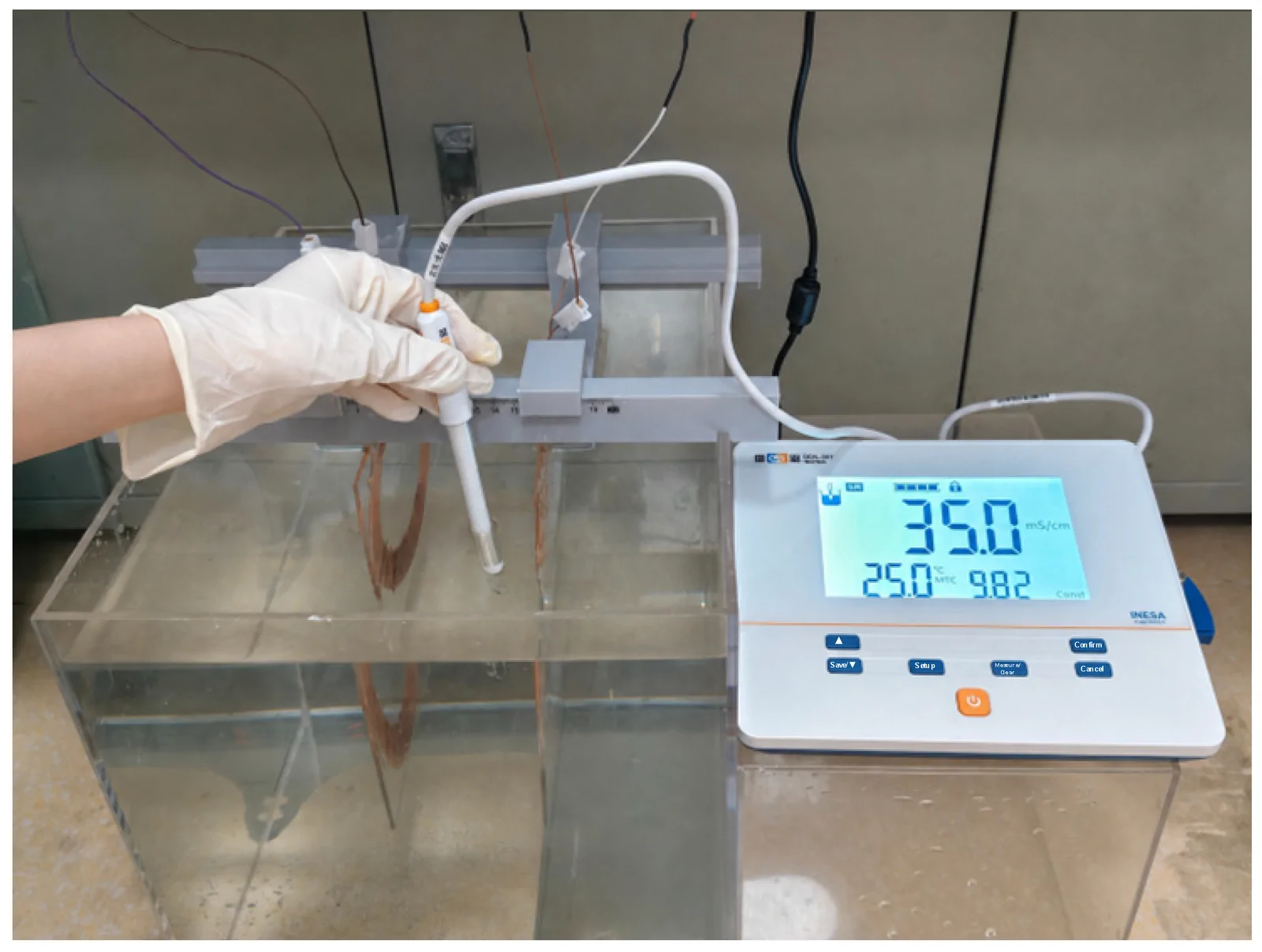
Conductivity Test Results (Taking 3.5 S/m as an Example).

In the underwater experiments, solutions of different conductivities are prepared using pure water and artificial sea salt to simulate various underwater environments. Specifically, the conductivity of the freshwater environment is set to 0.05 S/m (with reference to the conductivity data of actual rivers in China and the commonly adopted values in this field [39,40]). In addition, solutions with conductivities of 3 S/m, 3.5 S/m, 4 S/m, and 5.3 S/m are prepared to represent wireless power transfer scenarios at different seawater depths. During the experiments, a Shanghai Leici DDS-307 conductivity meter equipped with a DJS-10VTC conductivity electrode was used to monitor the conductivity. The conductivity was measured at 30-min intervals to ensure that it remained constant throughout the experiments. The test results are shown in Figure 21, taking 3.5 S/m as an example. Under these conductivity conditions, experiments on resonance frequency characteristics at different transmission distances are conducted.

**Figure 22:**
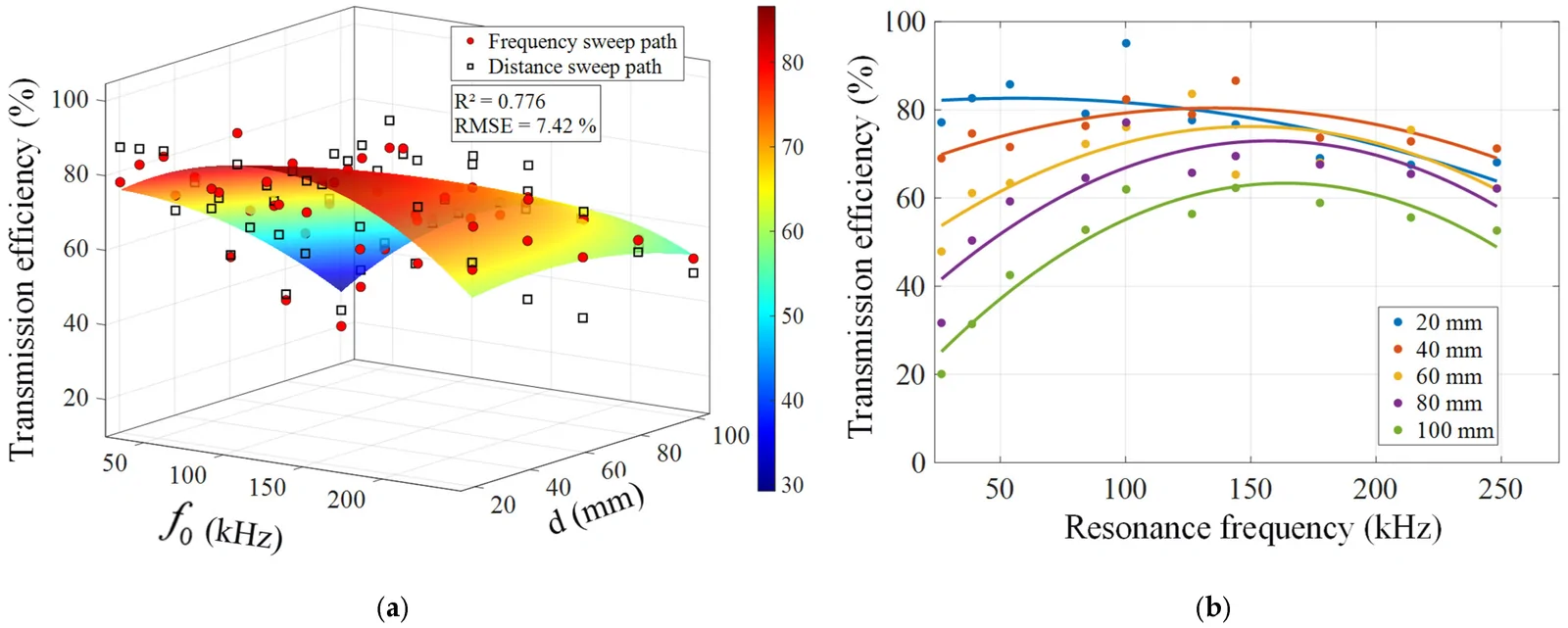
Experimental results of resonance frequency characteristics in freshwater: (**a**) three-dimensional fitted surface; (**b**) efficiency–frequency cross-sections.

The experimental results obtained in the freshwater environment are presented in Figure 22. It is observed that the transmission efficiency increases gradually to a peak and then decreases slightly as the resonance frequency rises. Moreover, the efficiency declines with increasing transmission distance.

**Figure 23:**
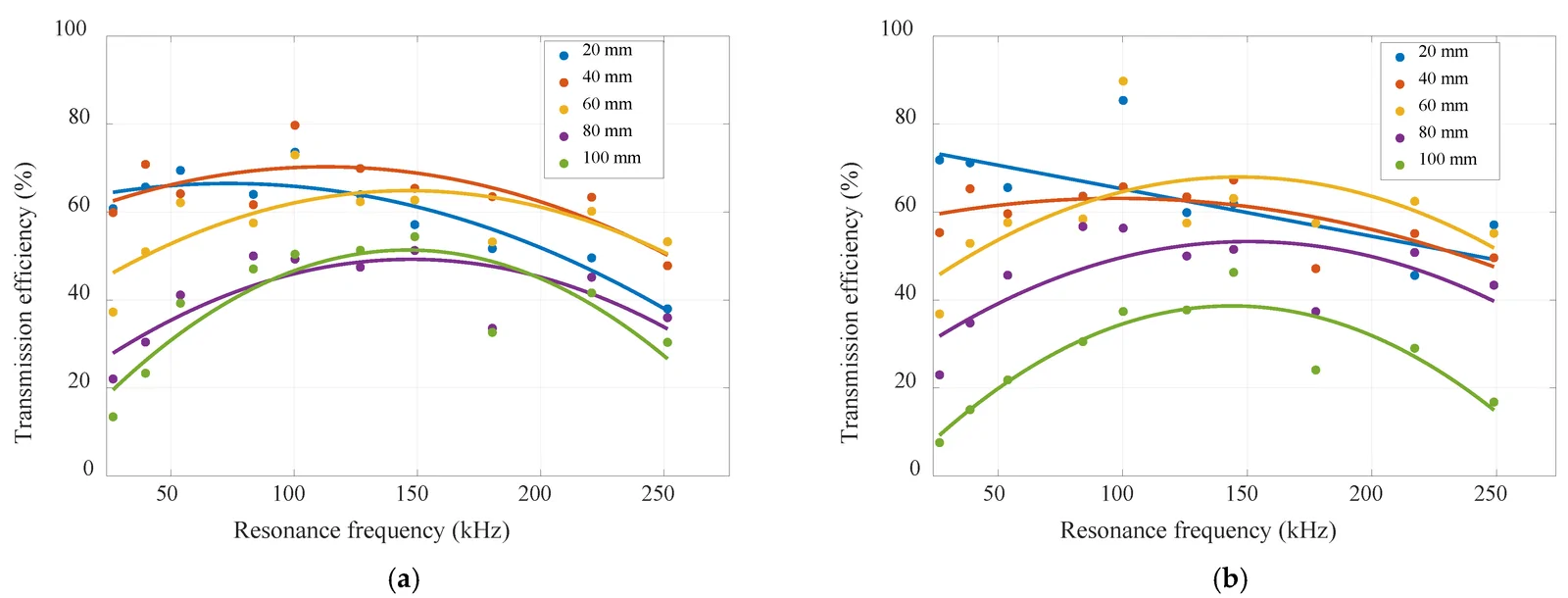

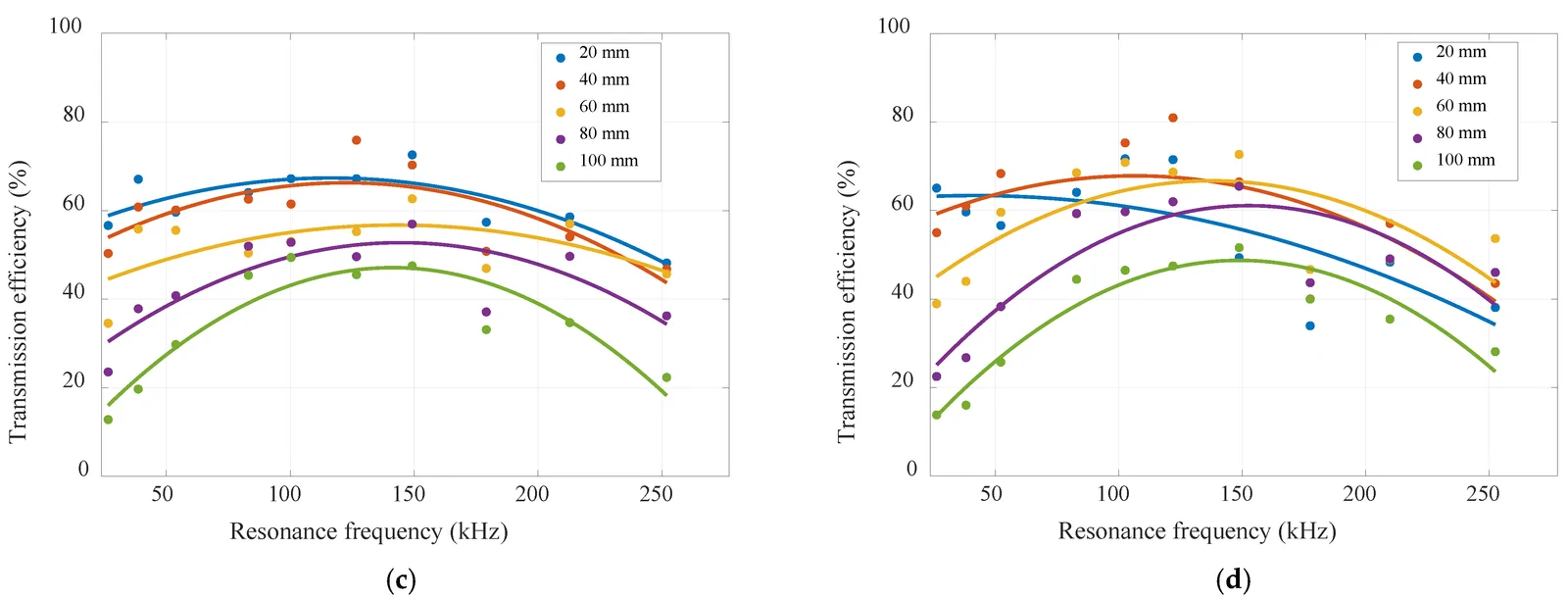
Transmission efficiency versus resonance frequency in underwater environments with different conductivity levels: (**a**) at 3 S/m; (**b**) at 3.5 S/m; (**c**) at 4 S/m; (**d**) at 5.3 S/m.

**Figure 24:**
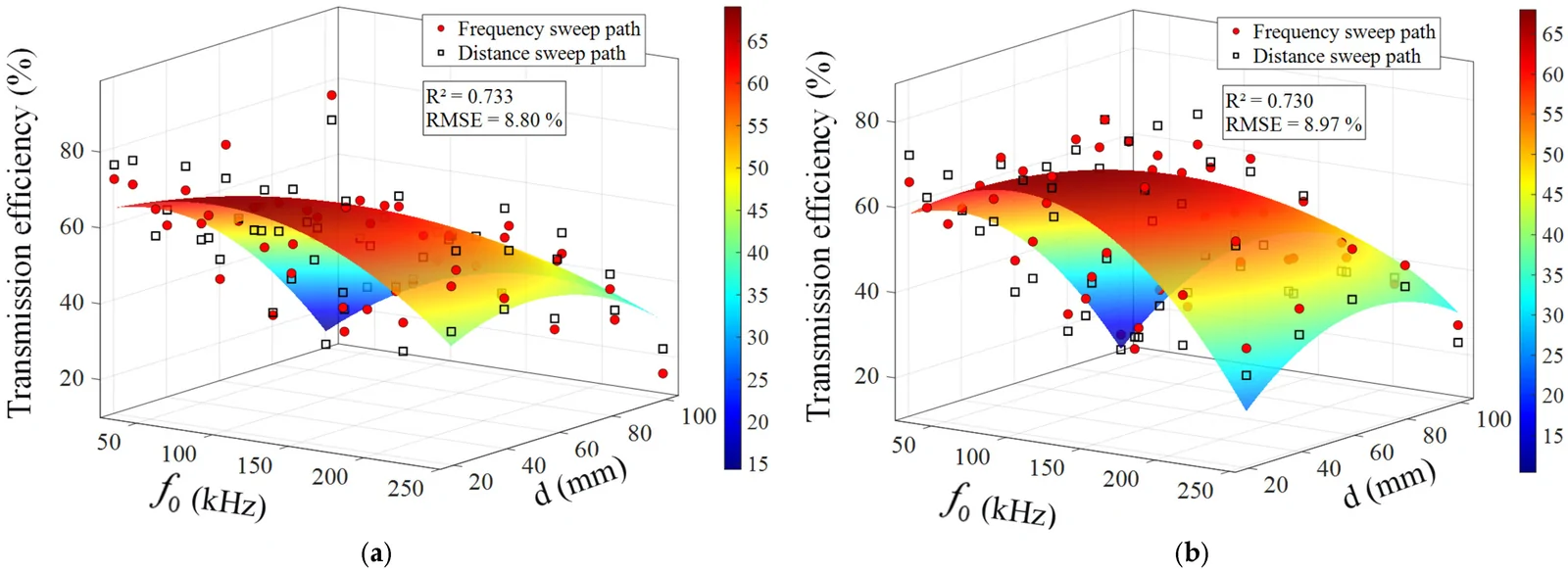
Experimental 3D fitting results in seawater: (**a**) at a conductivity of 3.0 S/m; (**b**) at a conductivity of 5.3 S/m.

Figure 23 and Figure 24 present the experimental results obtained in seawater with high conductivity. It is observed that, for each conductivity level and transmission distance, the efficiency first increases and then decreases as the resonance frequency rises. Peak efficiency is achieved at approximately 145 kHz, corresponding to the optimal resonant state. As the frequency deviates from this point, the efficiency declines. In addition, the overall transmission efficiency decreases as the conductivity increases, and the decline becomes more pronounced at higher conductivity levels. This is because higher conductivity leads to greater eddy current loss, which in turn increases system energy loss and reduces the transmission efficiency. Furthermore, the transmission efficiency decreases with increasing transmission distance across all conductivity levels. The experimental results are in good agreement with the previous theoretical and simulation analyses, further confirming that both the varying conductivity and the resonance frequency in underwater environments significantly affect the transmission performance of WPT systems.

In addition, the coefficient of determination for the underwater experimental fitting is approximately 0.7, and the RMSE is about 8 percentage points. Overall, the fitting can reasonably represent the trend of the experimental data with changes in frequency and transmission distance. It can therefore be used for intuitive analysis and trend description of the experimental results.

An RMSE analysis was performed to evaluate the error between the theoretical and experimental transmission efficiency values. The error analysis shows that the maximum RMSE error is 31.95%, and the error increases significantly at higher resonant frequencies. The sources of error can be attributed to the following factors: (1) the internal resistance of the coils increases rapidly with frequency, resulting in higher circuit loss; (2) the accuracy limitations of the capacitors, resistors, and measurement instruments; (3) conductivity is strongly affected by temperature, and the influence of temperature fluctuations cannot be completely eliminated even though conductivity is measured every 30 min during the experiments; and (4) errors introduced by the theoretical modeling of eddy current loss.

The main contribution of this study is the derivation of an explicit expression. The feasibility of the proposed model has been verified, although high-accuracy prediction has not yet been achieved. The trends observed in the experiments are consistent with the theoretical analysis, supporting the validity of the proposed analytical model.

## 5. Conclusions

This study develops an analytical estimation method and derives an explicit expression for evaluating eddy current loss in seawater and system transmission efficiency. The dependence of these quantities on key parameters is qualitatively analyzed. The resonance frequency characteristics of the MCR-WPT system under different conductivities are also investigated. The theoretical analysis reveals that the transmission efficiency is jointly influenced by coil parameters, transmission distance, and resonance frequency. Moreover, an optimal resonance frequency that maximizes the efficiency is identified.

Through numerical and finite element analysis, the relationship among transmission efficiency, resonance frequency, and conductivity is revealed. Eddy current loss increases with both resonance frequency and conductivity. In seawater environments with different conductivity levels, the transmission efficiency first increases and then decreases as the resonance frequency rises, while higher conductivity leads to lower efficiency. Finally, an experimental platform is constructed, and solutions with different conductivity levels are prepared for validation. The experimental results show good agreement with the theoretical and simulation analyses.

## Figures and Tables

**Table 1:** Values of Other Physical Quantities.

Physical Quantity	Value	Description
Relative Permittivity	81	The static relative permittivity of pure water at low frequencies is approximately 81 [36,37]. In this study, the resonance frequency used in the theoretical calculations is below 1 MHz, while that used in the experiments does not exceed 300 kHz. This frequency range is therefore consistent with the above assumption.
Permeability	4π × 10^−7^ H/m	The magnetic susceptibility of seawater is extremely low, on the order of 10^−5^. The frequency range considered in this study, below 1 MHz, is far lower than the response frequency associated with ionic magnetization mechanisms.
Temperature/Salinity	Characterized by Conductivity	Salinity is characterized by conductivity, and variations in conductivity are mainly caused by differences in temperature.
Pressure Effect	Neglected	In underwater WPT, doubling the pressure results in only an approximately 0.02% decrease in transmission efficiency. The effect of pressure on transmission efficiency is therefore negligible [38].

**Table 2:** Operating parameters of the MCR-WPT system.

Parameter	Symbol (Unit)	Value
Original inductance of the coil	L1, L2 (μH)	113.8
Inner radius of the coil	Rmin (mm)	70
Outer radius of the coil	Rmax (mm)	95.7
Number of turns of the coil	N1, N2	20
Coil pitch	w (mm)	0.3
Coil parasitic resistance	R1, R2 (Ω)	0.32
Wire diameter	2 r (mm)	1

**Table 3:** The detailed simulation parameters.

Parameter	Simulation Setting
Solution Type	Eddy Current
Computational Domain Dimensions	A cylinder with a base radius of 150 mm and a height of 350 mm
Waterproof Layer Thickness	4 mm
Waterproof Layer Material	Air
Relative Permittivity of Seawater	81
Excitation	1.5 A Current Excitation
Transmission Distance	100 mm

**Table 4:** Detailed Information on the Test Instruments.

Instrument Name	Model	Measurement Bandwidth	Measurement Accuracy	Operating Conditions
Load	15 Ω Resistor	N/A	N/A	N/A
Waveform Generator	RIGOL DG2102	1000 MHz	Vertical resolution: 16 bits; frequency stability: ±1 ppm; output jitter: as low as 200 ps; amplitude accuracy: ±(1% of setting ± 5 mV)	6 Vpp sinusoidal signal; frequency set according to the parameters of the WPT system
Power Amplifier	Aigtek ATA-101B	1 MHz	Output voltage zero-point drift: ≤±0.2 V	Gain set to 6 dB
Conductivity Meter	Shanghai Leici DDS-307	N/A	Minimum resolution: 0.001 μS/cm, automatically switched according to the measurement range; reference error of the electronic unit: ±1.0% FS	Electrode constant: 9.82

## Data Availability

The raw data supporting the conclusions of this article will be made available by the authors upon request.
